# Hydroxylases involved in terpenoid biosynthesis: a review

**DOI:** 10.1186/s40643-023-00656-1

**Published:** 2023-07-13

**Authors:** Zihan Zhang, Qing-Yang Wu, Yue Ge, Zheng-Yu Huang, Ran Hong, Aitao Li, Jian-He Xu, Hui-Lei Yu

**Affiliations:** 1 State Key Laboratory of Bioreactor Engineering, Shanghai Collaborative Innovation Centre for Biomanufacturing, East China University of Science and Technology, Shanghai, 200237 China; 2 CAS Key Laboratory of Synthetic Chemistry of Natural Substances, Shanghai Institute of Organic Chemistry, Chinese Academy of Sciences, Shanghai, 200032 China; 3 School of Life Sciences, State Key Laboratory of Biocatalysis and Enzyme Engineering, Hubei University, Wuhan, 430062 China

**Keywords:** Biooxidation, Cytochrome P450 monooxygenase, Hydroxylase, Natural products, Terpenoids

## Abstract

Terpenoids are pervasive in nature and display an immense structural diversity. As the largest category of plant secondary metabolites, terpenoids have important socioeconomic value in the fields of pharmaceuticals, spices, and food manufacturing. The biosynthesis of terpenoid skeletons has made great progress, but the subsequent modifications of the terpenoid framework are poorly understood, especially for the functionalization of inert carbon skeleton usually catalyzed by hydroxylases. Hydroxylase is a class of enzymes that plays an important role in the modification of terpenoid backbone. This review article outlines the research progress in the identification, molecular modification, and functional expression of this class of enzymes in the past decade, which are profitable for the discovery, engineering, and application of more hydroxylases involved in the plant secondary metabolism.

**Graphical Abstract:**

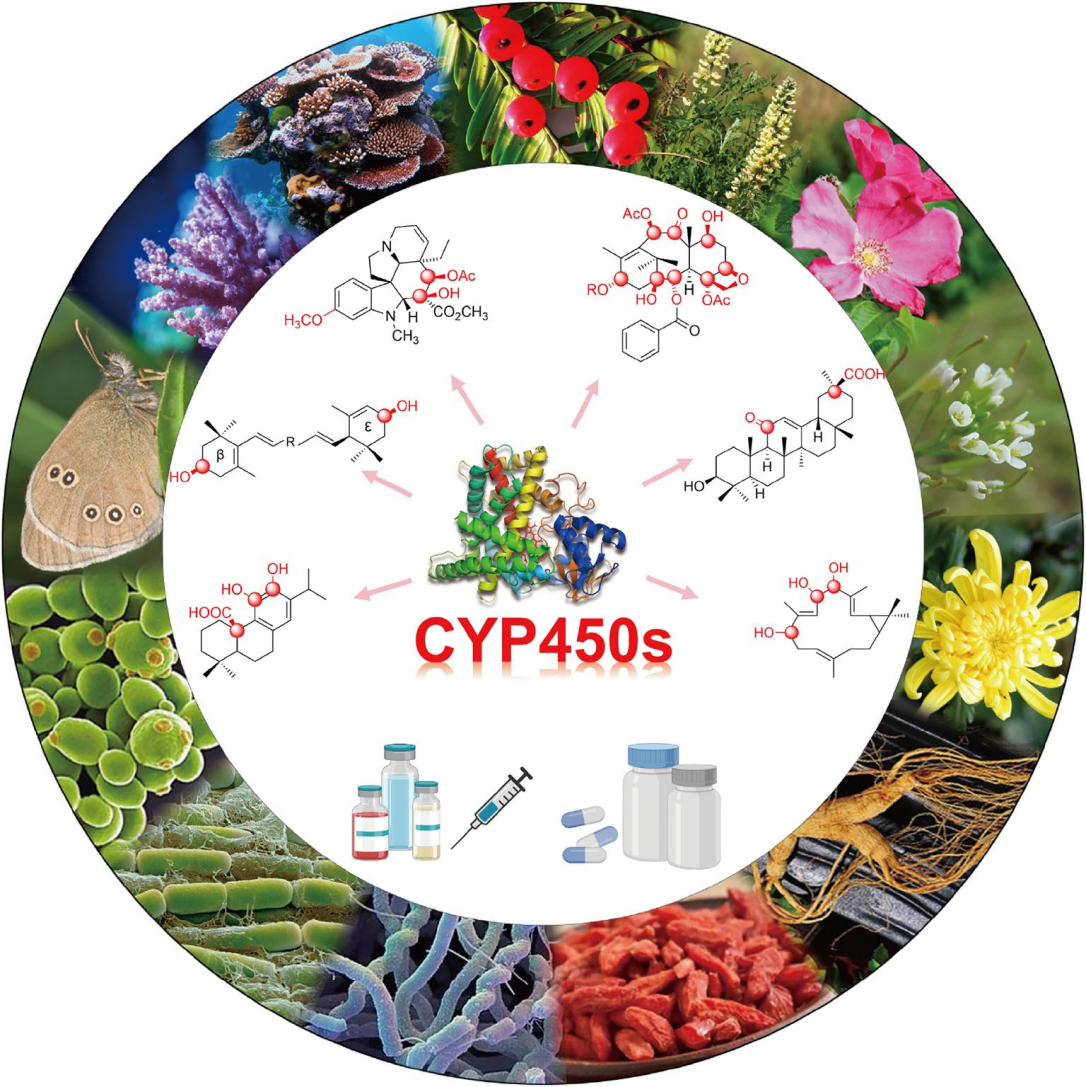

## Introduction

Terpenoids are the most diverse and abundant class of secondary metabolites among natural products, featuring tremendous structural and functional diversity with a series of significant pharmacological and biological activities (Chacon-Morales 2022). More than 80,000 terpenoids have been recorded in the Dictionary of Natural Products (http://dnp.chemnetbase.com) (Chen et al. 2020).

The extraordinary low content of these terpenoids in natural resources renders a direct extraction unsustainable. Moreover, chemical syntheses of complex terpenoids are generally received with the tedious multiple steps and extremely low overall yield. In addition, a large number of organic reagents are required for the above two approaches to ensure the extraction rate and product purity, posing serious circumstance destructions. In the past decade, the rapid developments in synthetic biology offer new possibilities for the efficient heterogeneous overproduction of terpenoids in microbial hosts, which can reduce the use of organic reagents and shorten the synthetic routes to a certain extent. At present, many kinds of terpenoids have been synthesized by synthetic biological methods, such as antimalarial compound artemisinin (Paddon and Keasling 2014), the precursor of famous anticancer drugs taxol (Ajikumar et al. 2010; Walls et al. 2021), and liver protecting ingredient glycyrrhizic acid (Sun et al. 2020).

Heterogeneous biosynthesis of terpenoids depends on the clarification of the corresponding metabolic pathways. In the biosynthetic pathway of terpenoids, two kinds of C5 building blocks, isopentenyl pyrophosphate (IPP) and dimethylallyl pyrophosphate (DMAPP), are cyclized or arranged to form different types of oligomers, such as geranyl pyrophosphate (GPP), farnesyl pyrophosphate (FPP), and geranylgeranyl pyrophosphate (GGPP). Then, a series of terpenoid skeletons are assembled under the catalysis of various cyclases. The subsequent modification processes of these skeletons contribute greatly to the structural diversity and pharmacological activities of terpenoids, which are mainly enabled by cytochrome P450 enzymes (CYPs) (Behrendorff and Gillam 2017).

CYPs are able to activate the inert C–H bond on the terpene backbone with high stereoselectivity, which keep been the bottlenecks to breakthrough in chemical synthesis. Although CYPs are capable to catalyze a variety of reactions, including hydroxylation, dealkylation, sulfur oxidation, epoxidation, deamination, desulfurization and peroxidation reactions, the most iconic reaction remains the insertion of hydroxyl or oxygen atoms into the C–H backbone to form the corresponding alcohols, which may be further oxidized to aldehydes or acids. These oxygen-containing groups are often necessary for the pharmacological activity of natural products. Harvesting the capacity of hydroxylases would be anticipated to greatly enhance the producibility of valuable natural products. However, some CYPs exhibit the low heterologous expression, poor stability, low catalytic activity, as well as the catalytic promiscuity of the extant CYPs, representing the bottlenecks of terpenoids heterologous biosynthesis.

This article reviews the various types of hydroxylases, especially cytochrome P450s, involved in the skeleton modifications of monoterpenes, sesquiterpenes, diterpenes, triterpenes, and tetraterpenes, and their corresponding oxidations. The challenges in the current research and corresponding measures are summarized. We expect that this summary can provide a guidance for the discovery of new terpenoid hydroxylases and the subsequent heterologous expression and modifications to exploit novel bioactive compounds for medicinal applications.

## Classification of terpenoid hydroxylases

The principle of terpenoid biosynthesis is using terpene synthase to unite the isoprene subunit chain to form monoterpenes, sesquiterpenes, diterpenes, triterpenes, and tetraterpenes according to the number of isoprene units (Huang et al. 2021). Terpenoid hydroxylases then catalyze the oxidation to activate the different carbon skeletons. Based on the different substrates, hydroxylases are classified into the following four categories.

### Monoterpene hydroxylases

Monoterpenes refer to terpenes and their derivatives containing two isoprene units in molecules, which are widely found in the secreted tissues of higher plants. Most of them have strong aroma and physiological activity and are important raw materials for the pharmaceutical and cosmetic industries. Monoterpene oxidases had been found in plants, microorganisms, and animals. According to the diversity of skeleton structure, monoterpene skeletons can be divided into acyclic and monocyclic forms (Corinna and Henrik 2013).

The CYP76 family occupies a large proportion in the monoterpene hydroxylases which catalyze the allylic hydroxylation of acyclic monoterpenes. For example, CYP76C1, CYP76C2, CYP76C4, and CYP76B6 can realize the allylic hydroxylation of linalool, geraniol, nerol, and citronellol (Hofer et al. 2014). CYP76C2 and CYP76C4 can also catalyze epoxidation of linalool at C-1 and C-2. CYP71B31 and CYP76C3, identified in *Arabidopsis thaliana* (*A. thaliana*), can also catalyze hydroxylation of linalool at multiple sites and epoxidation at C-1 and C-2 to produce four kinds of products, respectively (Ginglinger et al. 2013). Geraniol is the main agent of rose flavor essence and its derivatization may create novel flavor components. CYP76F45 can simultaneously catalyze allylic hydroxylation of monoterpene geraniol, sesquiterpene farnesol, and diterpene geranylgeraniol (Sintupachee et al. 2015). In recent years, P450s identified in beetles have attracted the attention of researchers. The pine bark beetle can convert myrcene into ipsdienol, the aggregation pheromone component. There are at least seven P450s in the organs or tissues of the mountain pine beetle. As the first cytochrome P450 known from insect, CYP6BH5 was identified from the liposome of leaf beetle and was expressed in Sf9 insect cells, and then catalyzed the hydroxylation of geraniol and its structural analogs, nerol and citronellol (Fu et al. 2019).

Limonene is a widely studied monocyclic monoterpene. Many alkane degrading microorganisms can catalyze *L*-limonene to produce perillyl alcohol. A limonene 7-hydroxylase CYP153A6 was isolated from the *Mycobacterium* SP.HXN-1500 strain (van Beilen et al. 2005). CYP750B1 and CYP76AA25 identified from *Thuja Plicata* can catalyze the allylic hydroxylation of dicyclic monoterpene (+)-sabinene to produce (+)-*trans*-sabinol which is the biosynthetic precursor of α-thujone. The substrate spectrum of CYP76AA25 is wide and also applicable to sesquiterpenoid farnesene and herbicide isoproturon (Gesell et al. 2015). CYP345E2 (Keeling et al. 2013), CYP6DE1 (Chiu et al. 2019a), CYP6DE3 (Nadeau et al. 2017), and CYP6DJ1 (Chiu et al. 2019b) were identified from mountain pine beetle and had been functional characterized to enable the allylic hydroxylation of α-pinene, β-pinene, limonene, and other monoterpenes to produce a variety of products with significant application value (Song et al. 2013). The difference is that CYP6DJ1 is specified to the two enantiomers of monocyclic monoterpene limonene to generate various epoxides and perillyl alcohol, while it has no catalytic activity for dicyclic monoterpene pinene and other terpinolenes.

**Fig. 1:**
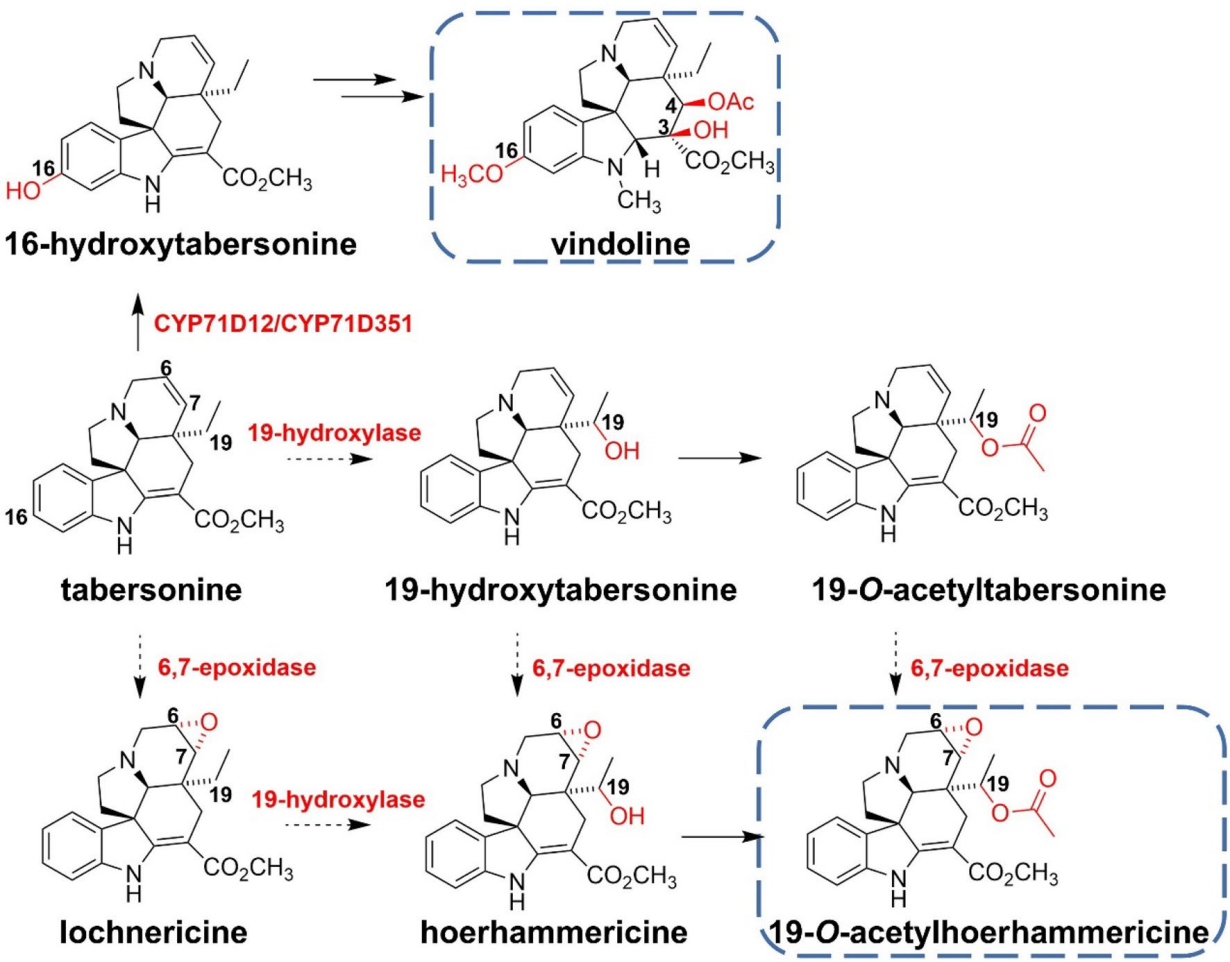
Proposed biosynthetic pathway of tabersonine-derived alkaloids (vindoline and 19-*O*-acetylhoerhammericine) in *C. roseus*

**Fig. 2:**
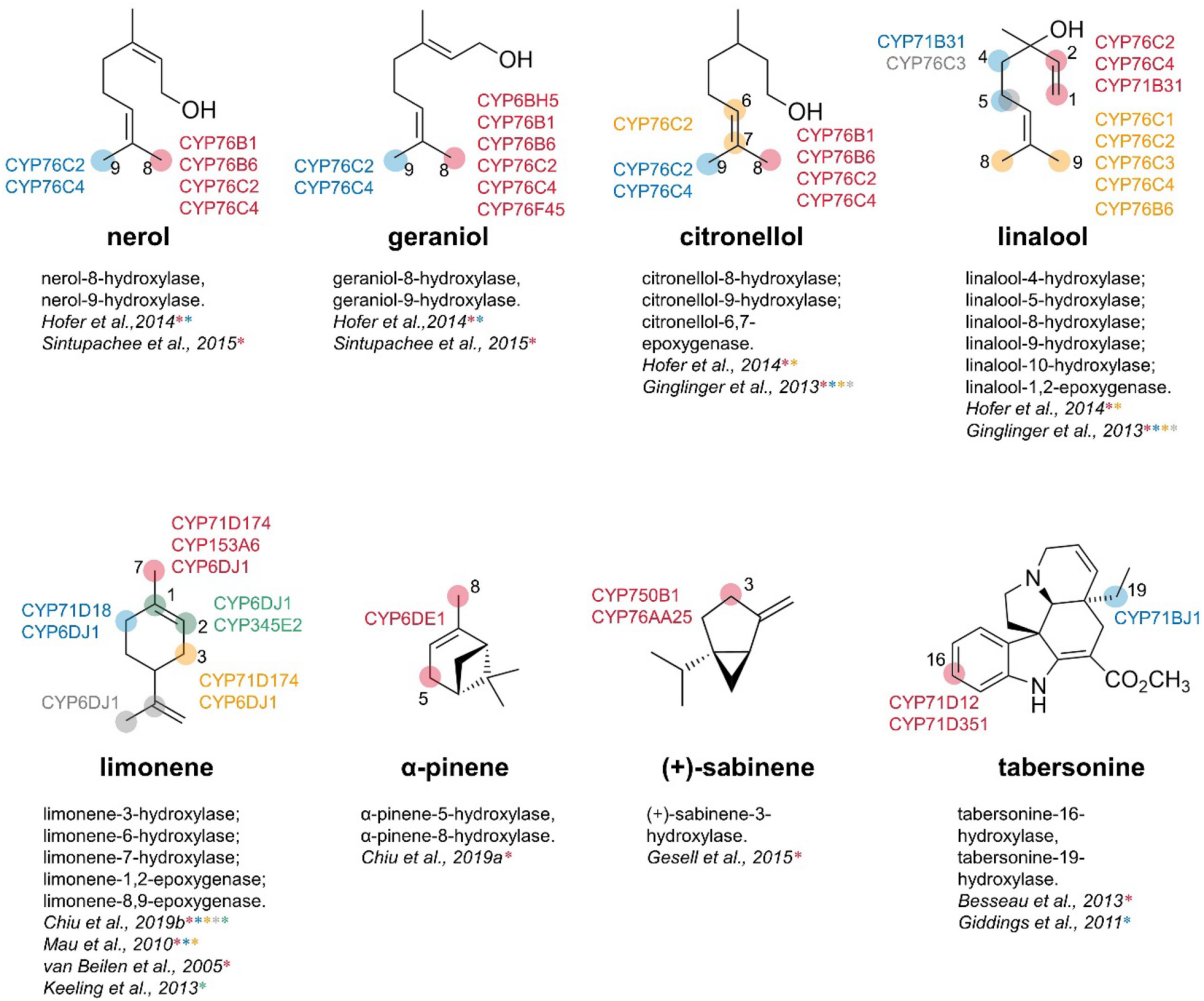
Monoterpene hydroxylases involved in this article

Vindoline is a kind of tabersonine-derived monoterpene indole alkaloid (MIA) from *Catharanthus roseus* (*C. roseus*), and its main role is to resist the ingestion of herbivores to protect plants. Vindoline serves as the precursor to vinblastine and vincristine which are used as antitumor drugs. CYP71D12 and CYP71D351 were found to hydroxylate the C-16 site of tabersonine with extremely high affinity and substrate specificity (Besseau et al. 2013). CYP71BJ1 promotes the hydroxylation of the C19 site in tabersonine to produce 19-hydroxytabersonine, which is then further epoxidized by 6,7-epoxidase to generate 19-*O*-acetylhoerhammericine (Giddings et al. 2011) (Fig. 1). Due to that CYP71BJ1 can also adopt lochnericine and tabersonine as substrates in vitro, the detailed pathway of hoerhammericine biosynthesis remains unclear (Fig. 2).

### Sesquiterpene hydroxylases

Sesquiterpenoids are natural terpenoids with higher boiling points than monoterpenes, which are polymerized from three molecules of isoprene. Most of their oxygen-containing derivatives have strong aroma and obvious biological activity. Sesquiterpene displays highly diverse branched skeletons, including mono-(Germacrene A, humulene, and zerumbone), bi-(β-caryophyllene, amorpha-4,11-diene, (+)-valencene, (+)-nootkatone), and tricyclic hydrocarbon skeletons (pentalenene, α-cedrene, and (+)-3(15)-cedren-4-ol) (Corinna and Henrik 2013).

**Fig. 3:**
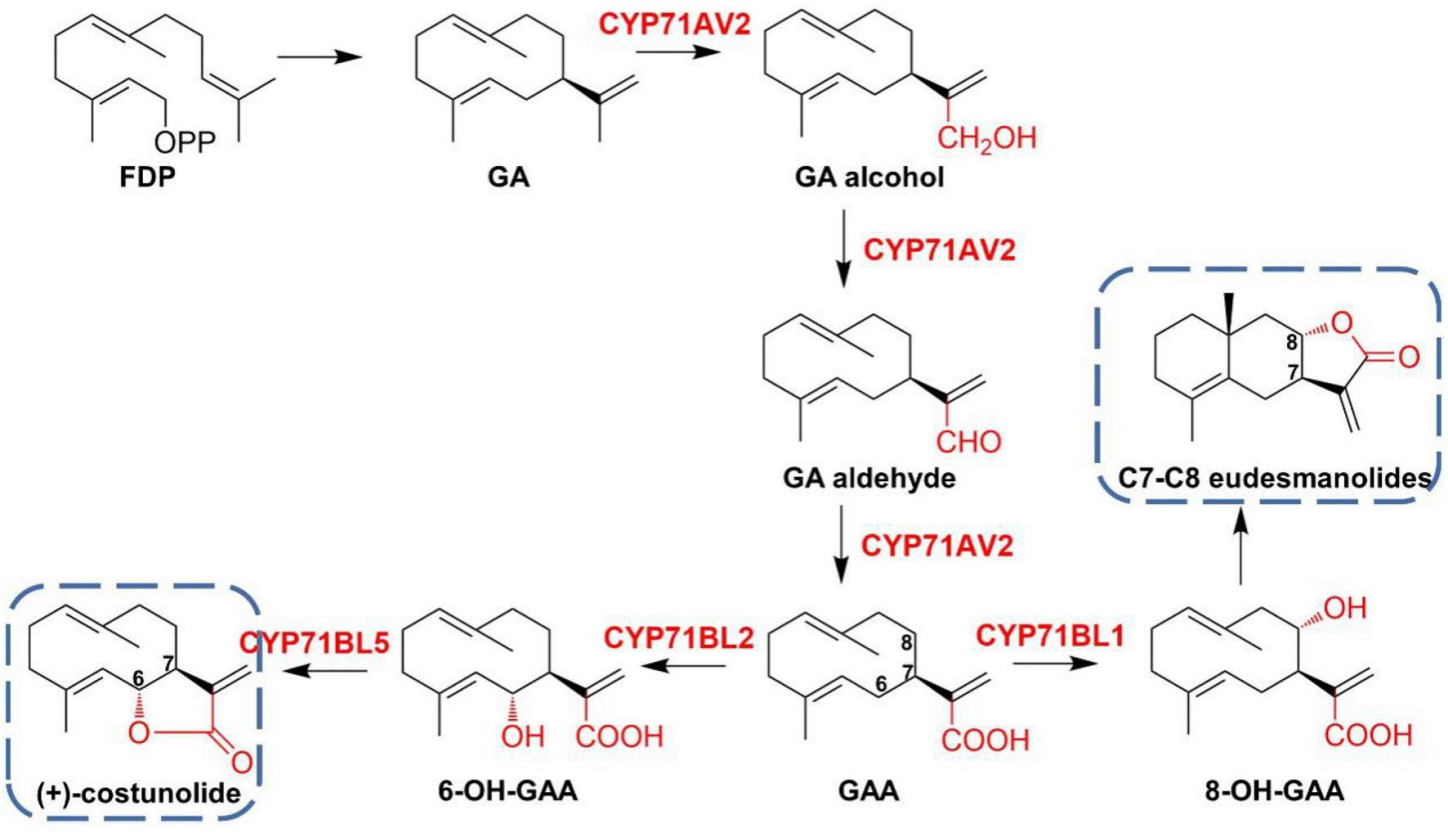
Proposed biosynthetic routes to C6–C7 and C7–C8 sesquiterpene lactones

Sesquiterpene lactones (STLs) are unique natural metabolites in *Asteraceae* plants. They can be used as anti-inflammatory, sedative, analgesic, anticancer, and anti-malaria drugs. Germacrene A (GA) and germacra-1(10),4,11(13)-trien-12-oic acid (GAA) are important monocyclic precursors to the synthesis of sesquiterpene lactones. CYP71AV2 has the function as GA oxidase, which promotes the oxidation reaction of the methyl group at C-12 of GA to generate GAA in three consecutive steps (Ramirez et al. 2013). Ikezawa et al. identified GAA-6-hydroxylase CYP71BL2 and GAA-8-hydroxylase CYP71BL1 from lettuce and sunflower, respectively (Ikezawa et al. 2011). Eljounaidi et al. isolated GA hydroxylase CYP71AV9 and GAA hydroxylase CYP71BL5 from *Cynara cardunculus* (Eljounaidi et al. 2014). Co-expression of CYP71AV9 together with GA synthase (GAS) during the biosynthesis of germacra-1(10),4,11(13)-trien-12-oic acid in yeast indicated that CYP71AV9 enabled the hydroxylation of position C-12 of GA, with alcohols and aldehydes generated under acidic condition and acids under buffer culture condition (Eljounaidi et al. 2014). The co-expression of CYP71BL5 and CYP71AV9 with GAS in *Nicotiana benthamiana* (*N. benthamiana*) leads to costunolide conjugates verified CYP71BL5 as the costunolide synthase (Fig. 3).

**Fig. 4:**
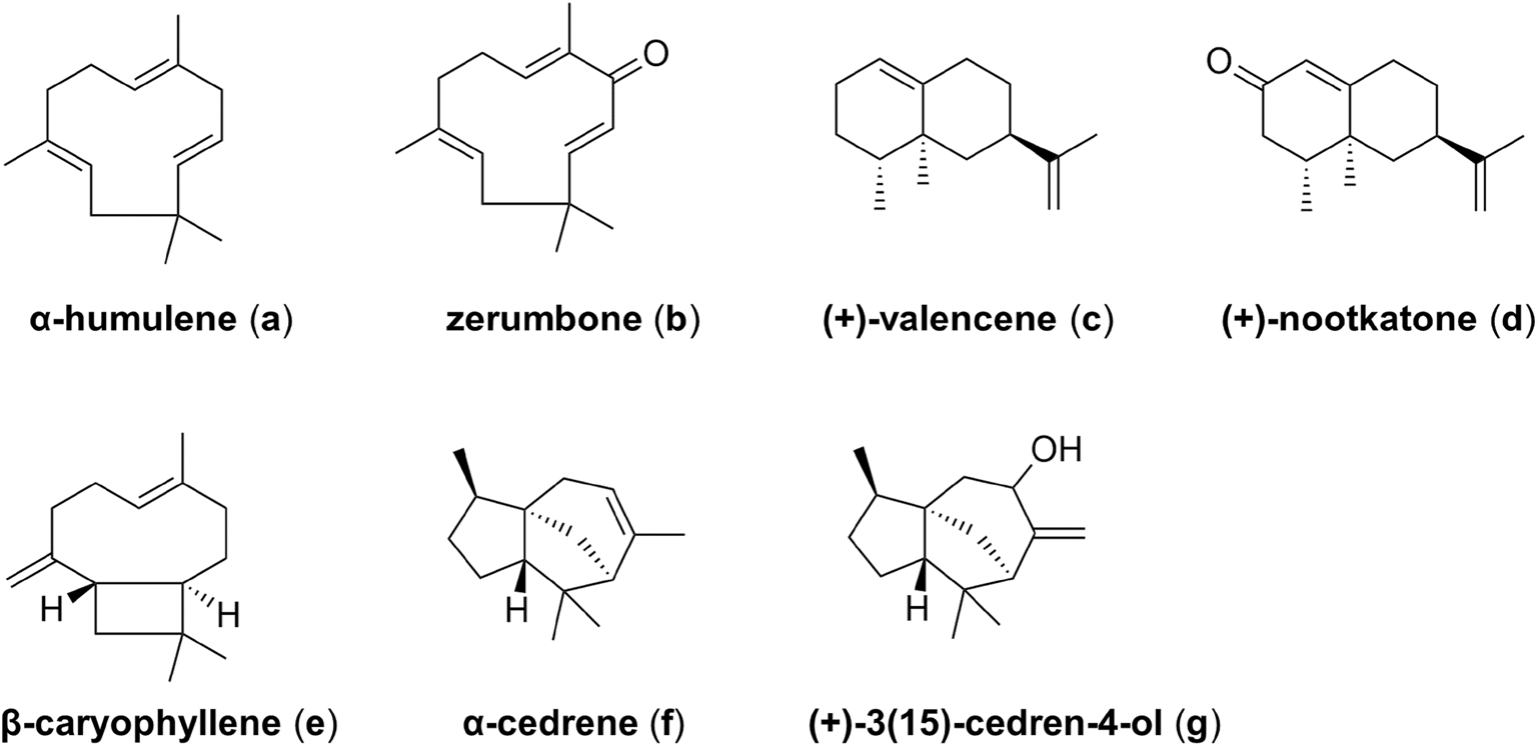
Substrate spectrum of CYP260A1 and CYP264B1

Monocyclic sesquiterpene zerumbone compounds exhibit anticancer, anti-inflammatory, and antinociceptive activities for potential application (Chien et al. 2016). The biosynthesis of zerumbone includes the selective hydroxylation of the basic skeleton α-humulene by cytochrome P450 to produce 8-hydroxy-humulene and then releases zerumbone through the alcohol dehydrogenase. Schifrin et al. isolated two sesquiterpene hydroxylases CYP260A1 and CYP264B1 from *Sorangium cellulosum* (*S. cellulosum*) Soce56 (Schifrin et al. 2015). CYP260A1 was found to catalyze the hydroxylation of monocyclic sesquiterpenes zerumbone (**b**) and bicyclic sesquiterpenes (+)-nootkatone (**d**), (+)-3(15)-cedren-4-ol (**g**) to form a variety of oxidation products. Compared to CYP260A1, CYP264B1 has a wider substrate tolerance, including monocyclic compounds zerumbone and α-humulene (**a**) and more complicated compounds, such as (+)-nootkatone, (+)-valencene (**c**), β-caryophyllene (**e**), and α-cedrene (**f**) (Fig. 4). It is worth noting that compared with CYP260A1, CYP264B1 has higher regioselectivity, with fewer kinds of products generated and a high proportion of specific product. The substrate promiscuity of these two hydroxylases is continually expanded and improved through molecular modification in order to produce the required high value-added products.

**Fig. 5:**
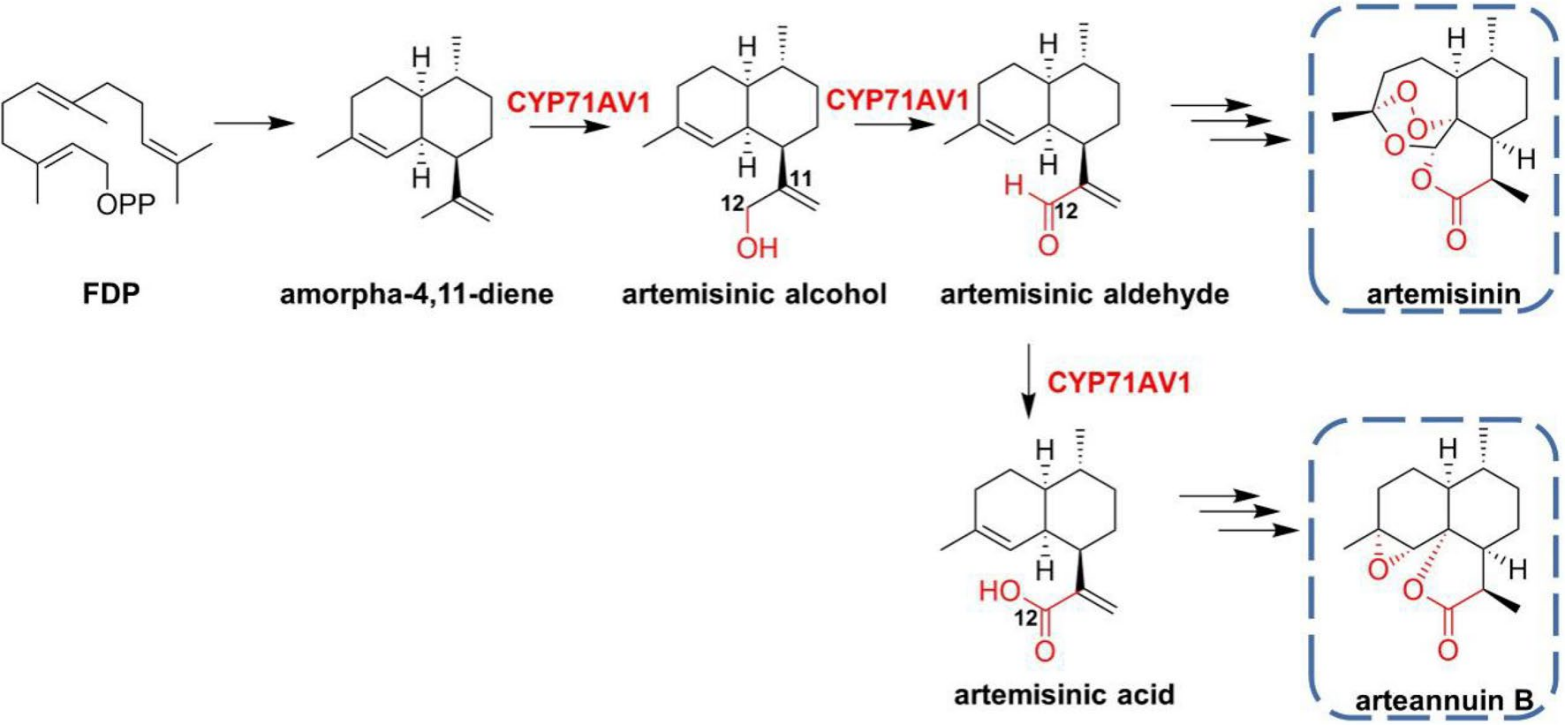
Biosynthetic pathway for sesquiterpene lactones artemisinin and arteannuin B

Bicyclic sesquiterpene artemisinin and derivatives represent the most effective class of antimalarial drugs. Its biosynthetic pathway has been largely elucidated in the past two decades. Firstly, farnesyl diphosphate is cyclized to amorpha-4,11-diene by amorphadiene synthase, and then CYP71AV1 catalyzes the three-step continuous hydroxylation of amorphadiene (Fig. 5). The regioselective oxidation at position C-12 produces artemisinol and that is further converted to artemisinic acid (Wang et al. 2013). The sequence identity of GA oxidase CYP71AV2 and amorpha-4,11-diene monooxygenase CYP71AV1 is as high as 86.7%, indicating that these two oxygenases may be evolved from the same ancestor.

**Fig. 6:**
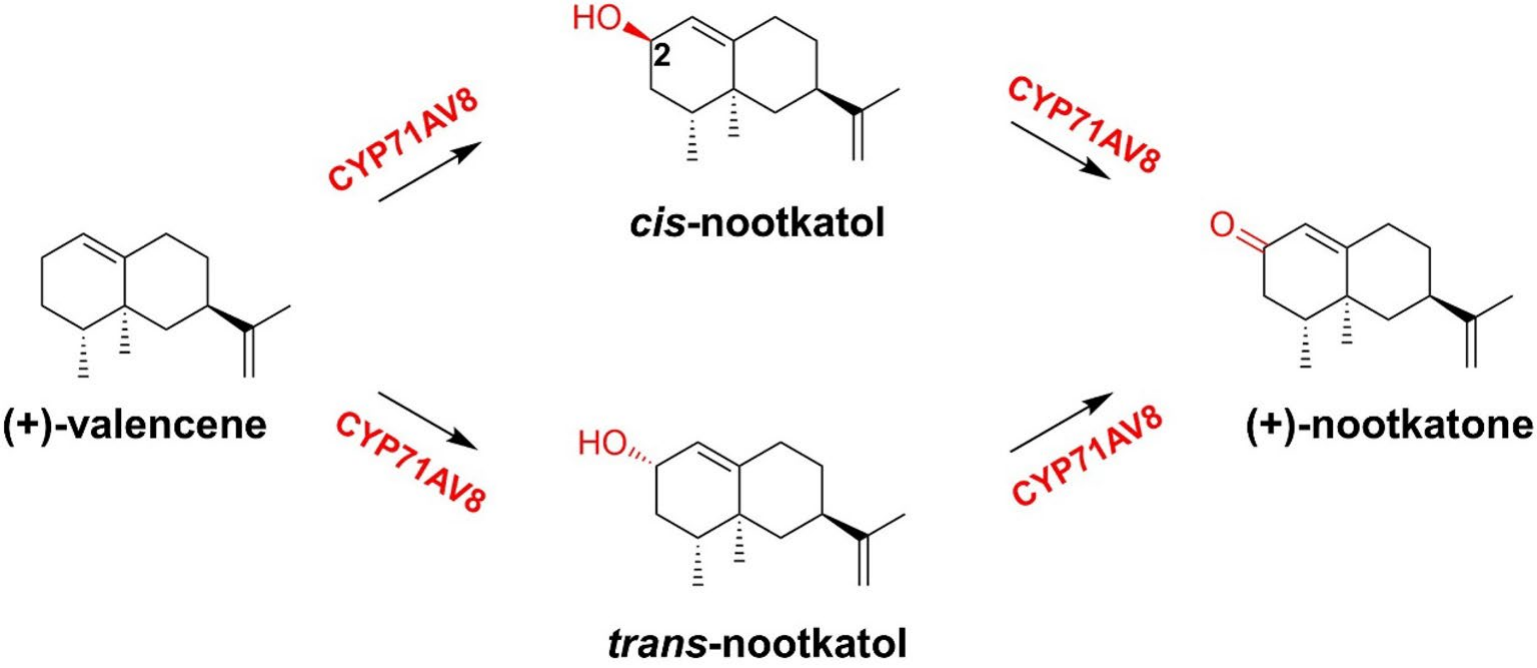
Conversion of (+)-valencene to (+)-nootkatone via *trans-* and *cis-*nootkatol catalyzed by CYP71AV8

Nootkatone, a bicyclic sesquiterpene compound with a grapefruit-like odor, is widely used in the food and fragrance industries. The high cost of natural extraction limits its industrial application. Cankar et al. screened out a new P450 protein CYP71AV8 with the sequence identity of 81% to amorpha-4,11-diene oxidase CYP71AV1. CYP71AV8 was able to catalyze the hydroxylation of (+)-valencene at the C-2 position to yield (+)-nootkatol, and a small part was further oxidized to (+)-nootkatone. With additional activities, like GA oxidase and amorphadiene oxidase, CYP71AV8 promotes the sequential oxidation of both at the C-12 position to the corresponding alcohols, aldehydes, and acids. This is the first P450 that has been proven to have the hydroxylation function of (+)-valencene (Cankar et al. 2011). Girhard et al. verified that CYP109B1 from *Bacillus subtilis* can also catalyze the sequential oxidation of valencene at the C-2 position to produce nootkatone (Girhard et al. 2010). Takase et al. used homologous sequencing to identify CYP71BE5, which catalyzes the hydroxylation of α-guaiene at C-2 to generate alcohol, and further oxidation produces (−)-rotundone. This enzyme can also catalyze the hydroxylation of (+)-valencene at C-2 to yield β-nootkatol (Takase et al. 2016). Gavira et al. also isolated CYP71D51V2 from *Saccharomyces cerevisiae* (*S. cerevisiae*) to enable the oxidation of (+)-valencene to (+)-nootkatol as a major product (Gavira et al. 2013) (Fig. 6).

**Fig. 7:**
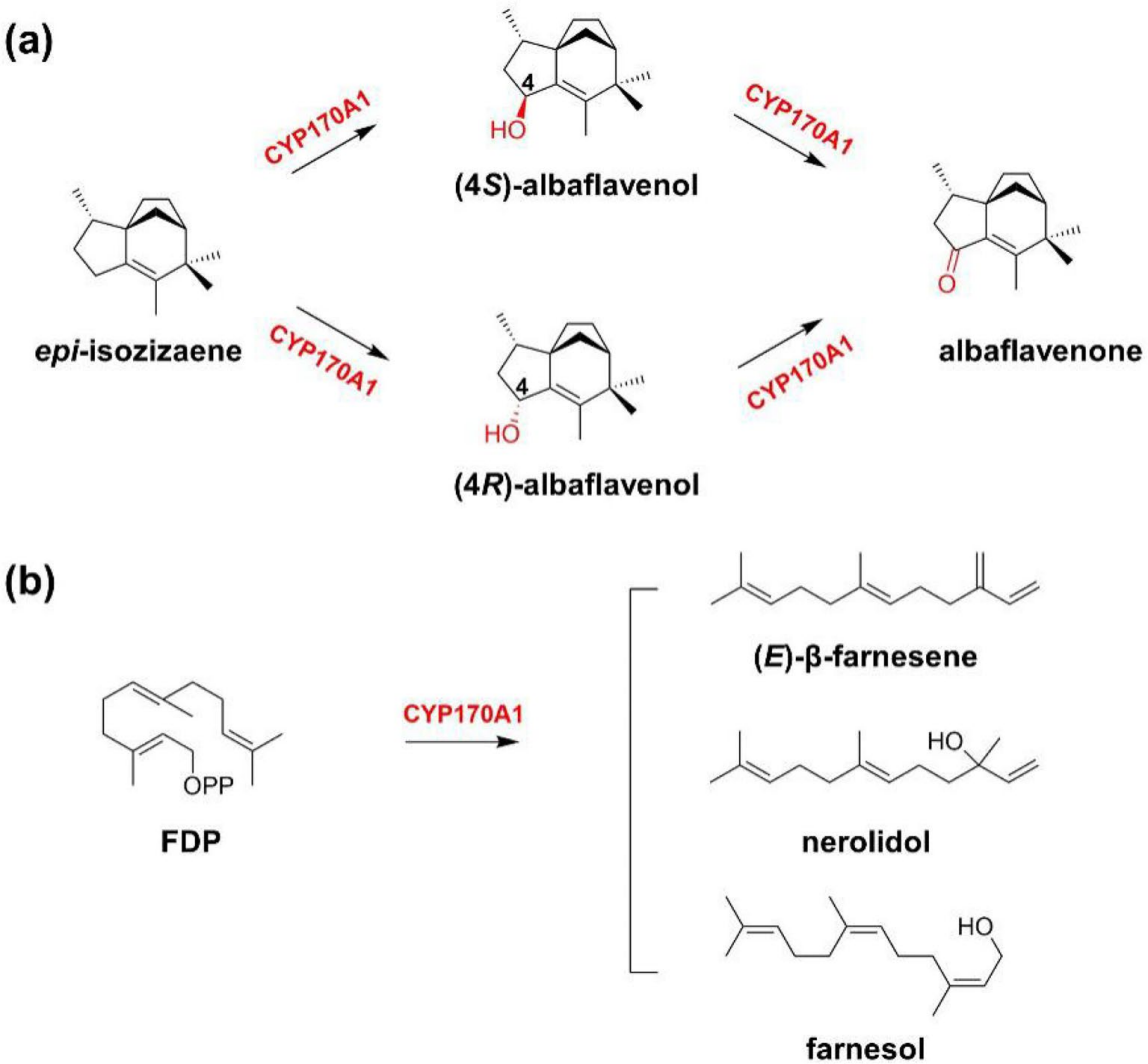
Bifunctional CYP170A1 with two different active sites. **a** Allylic oxidation of epi-isozizaene; **b** farnesyl diphosphate into acyclic sesquiterpene farnesene, farnesol, and nerolidol

**Fig. 8:**
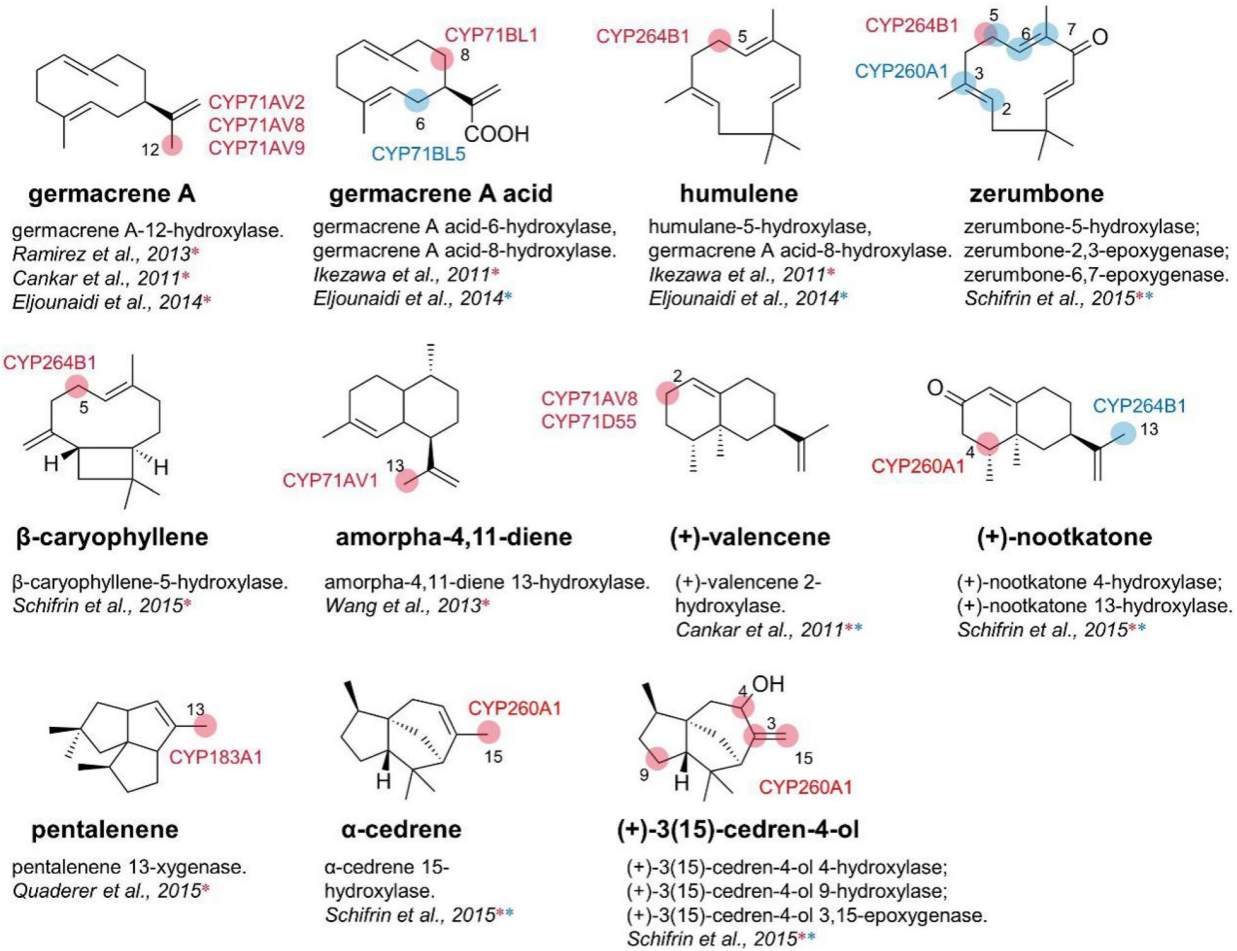
Sesquiterpene hydroxylases involved in this article

Members of the genus *Streptomyces* produce a variety of biologically active secondary metabolites, including most of the antibiotics widely used in medicine. A few of terpene hydroxylases were identified in the gene cluster of antibiotics biosynthesis. Quaderer et al. found CYP183A1 in the gene cluster for the biosynthesis of the tricyclic sesquiterpene antibiotic pentalenolactone catalyzes the hydroxylation of pentalenene at the allylic position to generate pentalen-13-ol, which is then gradually oxidized to pentalen-13-al, the precursor toward antibiotic pentalenolactone (Quaderer et al. 2006). Zhao et al. identified gene of CYP170A1 in *Streptomyces coelicolor* A3(2) and confirmed that CYP170A1 catalyzes the two-step continuous allylic oxidation of *epi*-isozizaene to generate the sesquiterpene antibiotic albaflavenone (Zhao et al. 2008) (Fig. 7). CYP170A1 was also found to convert farnesyl diphosphate (FDP) into acyclic sesquiterpene farnesene and farnesol. Enzymology and structural data clearly showed that CYP170A1 has active sites of farnesene synthase, which make it perform the additional terpene synthase activity in the presence of divalent cations (Zhao et al. 2009) (Fig. 8).

### Diterpene hydroxylases

Diterpenes are higher terpenoids polymerized from four molecules of isoprene. Due to their large molecular weight and poor volatility, diterpenes mostly exist in nature in the form of resins and lactones. There are very few acyclic and monocyclic diterpenoids in plants, mainly bicyclic and tricyclic diterpenoids, especially numerous oxygen-containing derivatives.

**Fig. 9:**
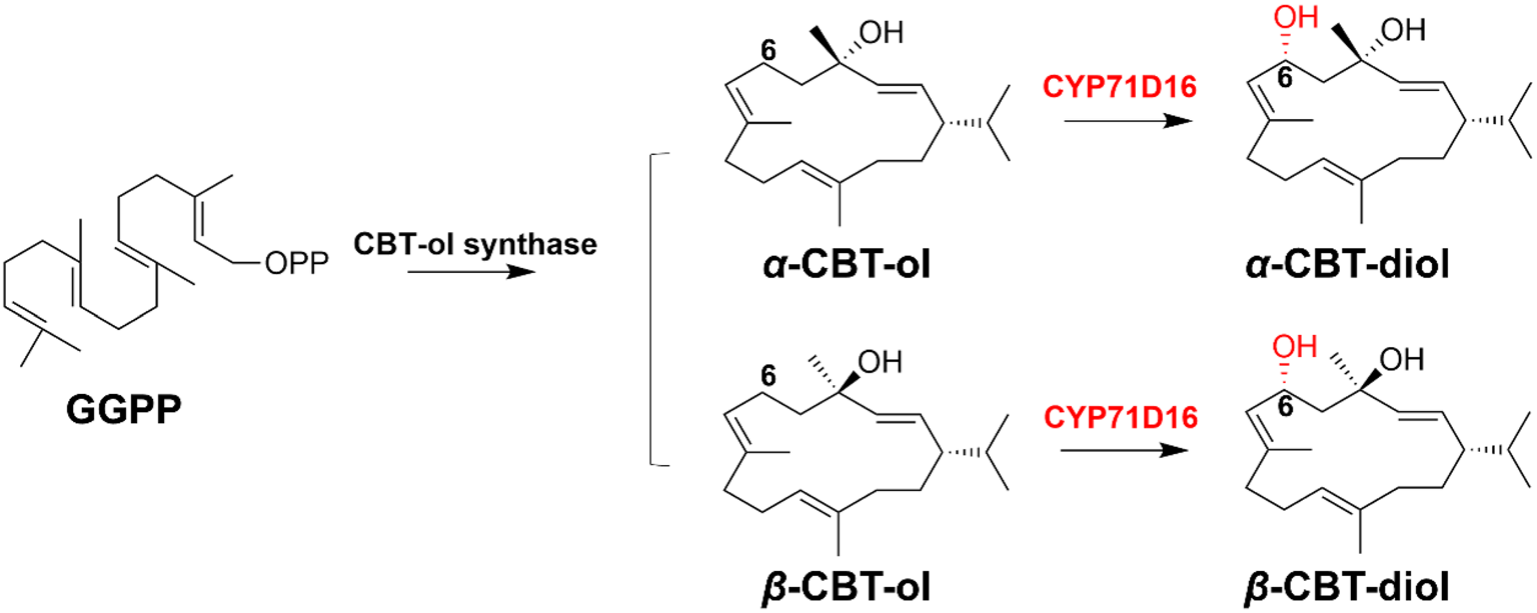
Biosynthetic pathways of CBT-ol and CBT-diol in tobacco. *GGPP* geranylgeranyl pyrophosphate

Cembranoids contain a large group of 14-membered macrocyclic diterpenoids whose types, positions, and stereochemical properties of their oxygen function are different, which are mainly derived from tobacco and corals. Cembranoids have a variety of biological activities, including antitumor and neuroprotective effects (Schrepfer et al. 2020). There exist narrow reports on CYP450s involved in their biosynthesis pathway. Wang et al. isolated the gene of CYP71D16 from trichome glands of tobacco using PCR-based complementary cDNA subtraction strategy. CYP71D16 catalyzed the hydroxylation at C-6 of cembratriene-ol (CBT-ol) to produce cembratriene-diol (CBT-diol), which is a key diterpene in the biosynthesis of the other cembranic compounds (Wang and Wagner 2003) (Fig. 9).

**Fig. 10:**
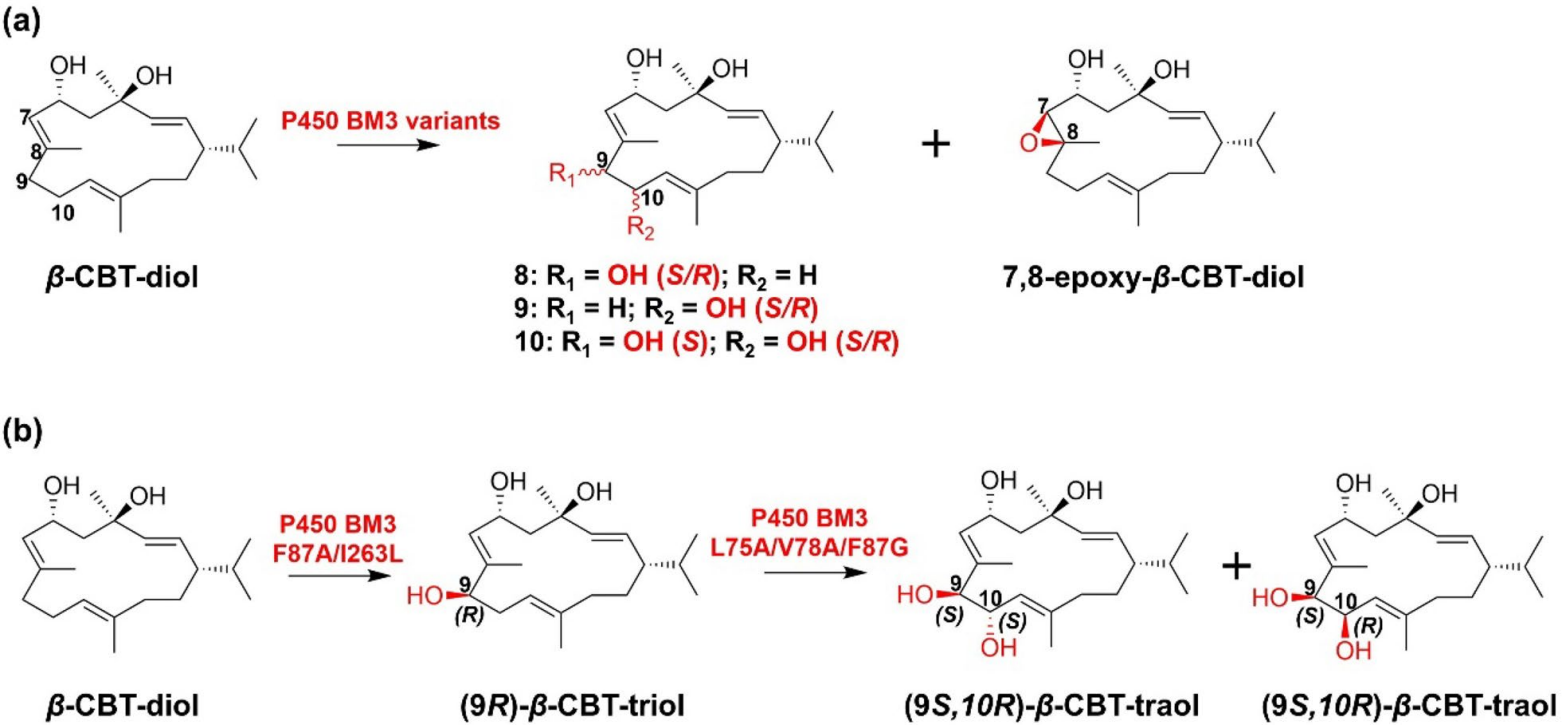
**a** Hydroxylation of *β*-CBT-diol catalyzed by P450 BM3 variants; **b** one-pot two-step hydroxylation of *β*-CBT-diol to form *β*-CBT-traol catalyzed by P450 BM3 variants. **8**: (9R/S)-*β*-CBT-triols; **9**: (10R/S)-*β*-CBT-traols; **10**: (9S,10R/S)-*β*-CBT-traols

By constructing P450 BM3 mutant library, Le-Huu et al. obtained a double P450 BM3 mutant F87A/I263L oxidize *β*-CBT-diol at C-9 with 100% regioselectivity and 89:11 diastereomeric ratio. A triple mutant L75A/V78A/F87G oxidize *β*-CBT-diol at C-10 with 97% regioselectivity and the diastereomeric ratio was 74:26 (Le-Huu et al. 2015). Following research showed that the triple mutant L75A/V78A/F87G can also hydroxylase C-10 of (9*R*)-*β*-CBT-triol to form (9*S*,10*S*)-*β*-CBT-traol and (9*S*,10*R*)-*β*-CBT-traol with diastereomeric ratio of 90:10 (Le-Huu et al. 2016) (Fig. 10).

The products derived from the casbene and the related derivatives are part of the most representative bicyclic diterpenes, exhibiting significant antitumor and antiviral activities. King et al. obtained casbene synthases and various P450s belonging to CYP726A through gene cluster mining. CYP726A14, CYP726A17, or CYP726A18 from *Ricinus communis* (*R. communis*) can catalyze the 5-oxidation of casbene to 5-keto-casbene and 5α-hydroxy-casbene. CYP726A16 was not able to functionalize casbene. However, by co-expressing casbene synthase, casbene-5-oxidase, and CYP726A16, 5-keto-7,8-epoxy-casbene was able to detected, indicating that the conversion of casbene to 5-keto-casbene is a linear step in the diterpenoid biosynthetic pathway. Interestingly, CYP726A15 derived from the same gene cluster can catalyze the oxidation of the C-5 position of neocembrene in two consecutive steps to produce 5-keto-neocembrene. Additionally, CYP726A19 from *Euphorbia peplus* (*E. peplus*) has the function of casbene 5-oxidase to give the same products as that used by CYP726A14 with a different proportion of the hydroxy- and the keto-products (King et al. 2014). The mature seeds of *Euphorbia lathyris* L. contain many macrocyclic diterpenes. Through transcriptome analysis of the mature seeds of *Euphorbia lathyris* L. and intracellular functional characterization, Luo et al. discovered two P450s function as regiospecific casbene hydroxylases, CYP71D445 and CYP726A27, that can catalyze the continuous two-step oxidation of C-9 and C-5 of casbene, respectively.

**Fig. 11:**
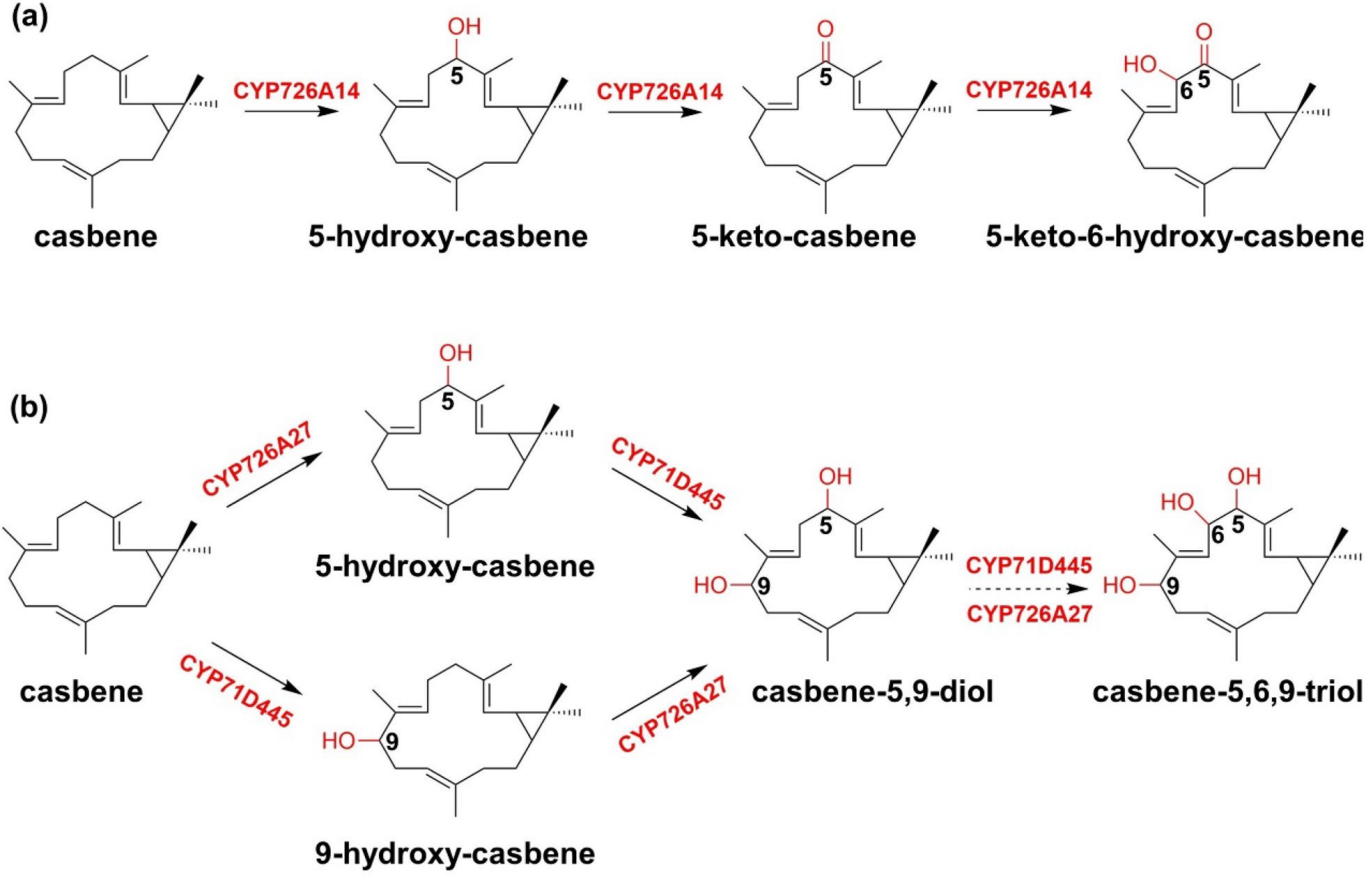
**a** Reactions of casbene catalyzed by CYP726A14 and potential role in castor bean diterpenoid biosynthesis. **b** Proposed pathway for the conversion of casbene to casbene-5,6,9-triol through enzymatic hydroxylations catalyzed by CYP71D445 and CYP726A27

Some of these enzymes are bifunctional enzymes. The initial product by CYP726A14 was characterized to be 5-hydroxy-casbene, which was subsequently oxidized to 5-keto-casbene and 5-keto-6-hydroxy-casbene with further hydroxylation (Boutanaev et al 2015). The combined in vitro assays of CYP71D445 and CYP726A27 demonstrated that the two P450s act as bifunctional enzymes, thereby clarifying their shared responsibility with a partial functional redundancy for catalyzing oxidation at C-6. Multifunction of these hydroxylases plays an important role in the diterpene modification leading to a great structural diversification (Fig. 11).

One of the representative tricyclic diterpenoids is carnosic acid and its related derivatives derived from the *Lamiaceae* plants. Those diterpenoids exhibit a variety of effects, including acting as anti-HIV and anticancer agents. The cyclized product miltiradiene from GGPP by the diterpene synthase is then oxidized to abietatriene and finally hydroxylated by a series of effective P450s, such as CYP76AH1 from *Salvia miltiorrhiza* (*S. miltiorrhiza*), CYP76AH4–7 from *Salvia fruticosa* (*S. fruticosa*) and CYP76AH22–24 from *Rosmarinus officinalis* (*R. officinalis*). With the initial name as ferruginol synthases (FSs), these P450s can hydroxylate dehydroabietadiene or abietatriene at C-12 to produce ferruginol and then play the role of the ferruginol 11-hydroxylase (HFSs) to generate 11-hydroxyferruginol (Bozic et al. 2015; Scheler et al. 2016; Zi and Peters 2013). Moreover, CYP76AK6–8 from *R. officinalis* and *S. fruticosa* can perform the three-step continuous oxidation on the C-20 position of CYP76AK6–8 to give carnosic acid. In addition, CYP76AK6–8 have the catalytic activity of oxidation on a variety of substrates, such as a direct oxidation of miltiradiene at the C-20 position to yield miltiradiene 20-ol. The discovery of a series of CYP450 genes involved in the biosynthetic pathway of carnosic acid was realized by the heterologous synthesis in yeast, providing an elegant example for the de novo synthesis of carnosic acid in other organisms, such as plants (Scheler et al. 2016).

**Fig. 12:**
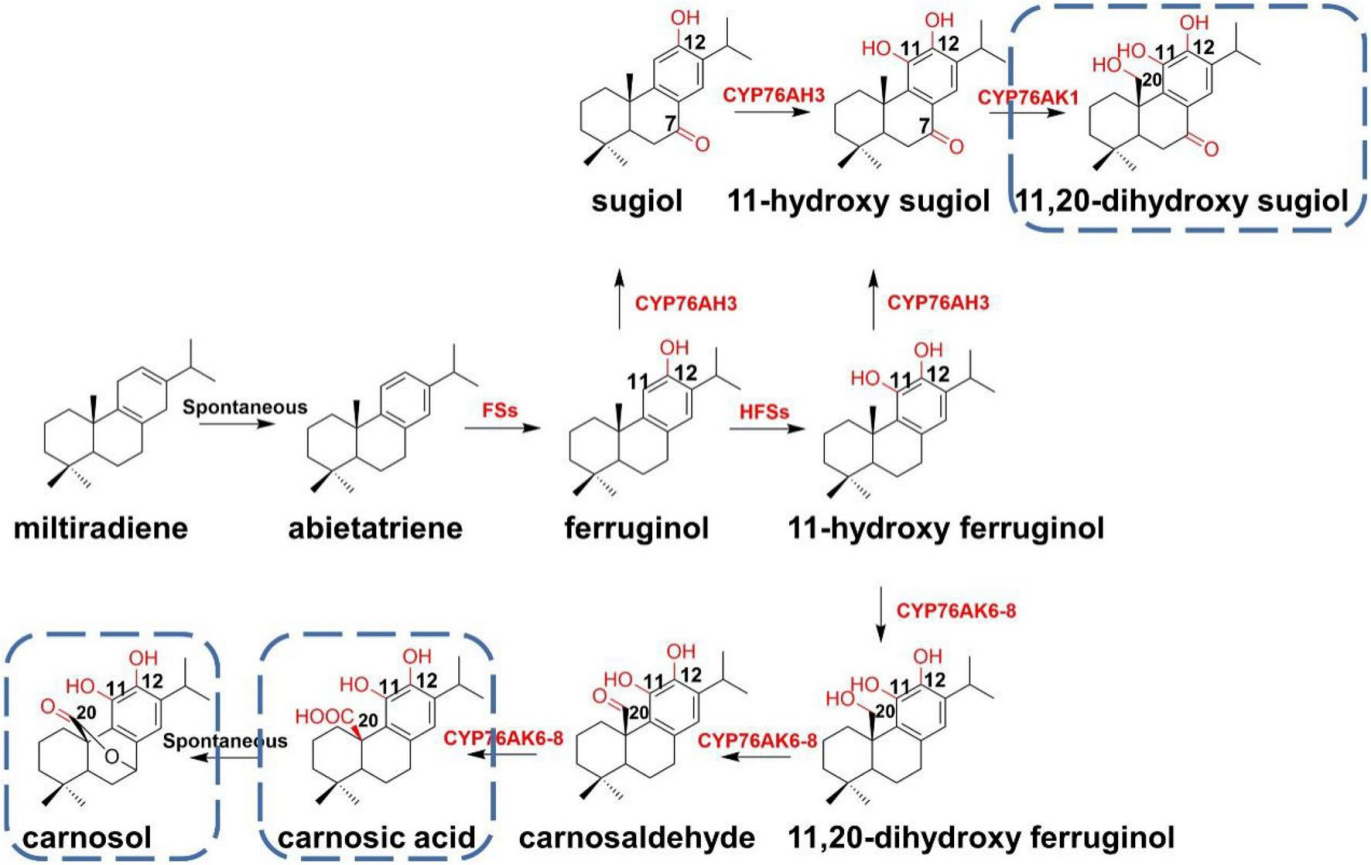
Proposed partial biosynthetic pathway of carnosic acid, carnosol and 11,20-dihydroxy sugiol. FSs: ferruginol synthases, including CYP76AH1, CYP76AH3, CYP76AH4–7, and CYP76AH22–24. HFSs: 11-hydroxyferruginol synthases

The promiscuity of some oxidases involved in this pathway leads to a dispersion of metabolic flow. CYP76AH3 can catalyze the oxidation of ferruginol at positions of C-11 and C-7 to deliver two intermediates, 11-hydroxy ferruginol, and 11-hydroxy sugiol, with no strict order of hydroxylation. CYP76AK1 was characterized to continue the transformation of the two intermediates produced by CYP76AH3, and hydroxylation of the C-20 position to form 11,20-dihydroxy ferruginol and 11,20-dihydroxy sugiol as the endpoint. Among them, the C-11 and C-12 hydroxyl groups of 11,20-dihydroxy ferruginol can be spontaneously oxidized to form the carbonyl groups in 10-hydroxymethyl tetrahydromiltirone (Guo et al. 2016). The catalytic promiscuity of CYP76AH3 and CYP76AK1 resulted in a variety of intermediates within the metabolic process (Fig. 12).

Resin acid is an important defensive component of conifers and has a protective effect against rapidly evolving pests and pathogens. A variety of diterpene cyclases in conifers catalyze GGPP to generate different products, including pimaradiene, isopimaradiene, sandaracopimaradiene, and dehydroabietadiene. CYP720B1 from *Pinus taeda* (*P. taeda*) and CYP720B4 from *Picea sitchensis* (*P. sitchensis*) can oxidize the C-18 sites of the above four substances in three consecutive steps to release the corresponding resin acids. The sequence identity of these two P450s is 86%, and the sequence similarity is 95%. Among them, CYP720B4 expressed in *Escherichia coli* (*E. coli*) and engineered yeast shows that its hydroxylation activities on multiple substrates produce various oxidized diterpenes (Hamberger et al. 2011).

**Fig. 13:**
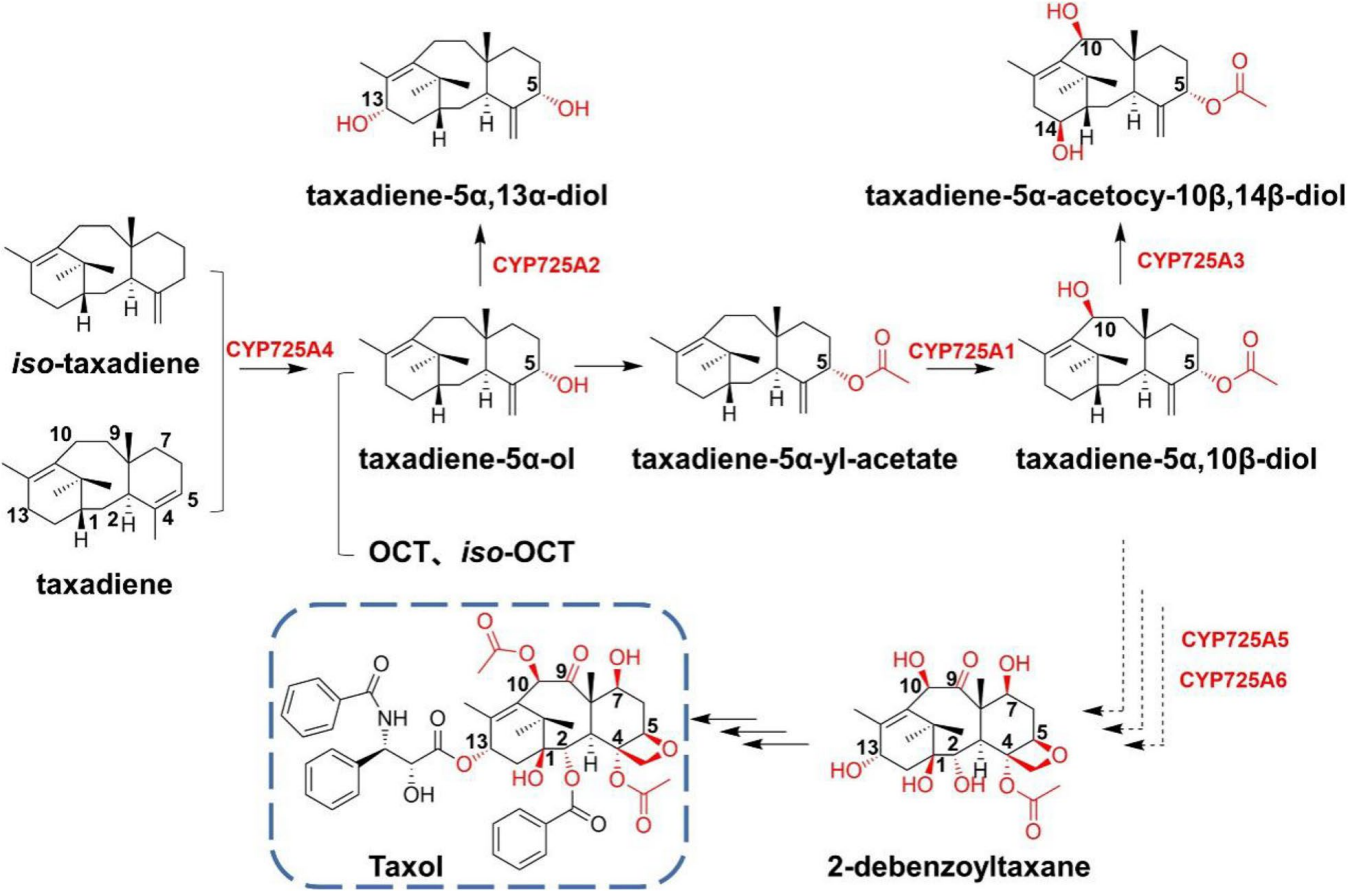
The complex skeleton modifications in the biosynthetic pathway of taxol, especially the hydroxylation at the various sites of taxane core skeleton

Taxol (Paclitaxel®) has low toxicity and potent anticancer activity and has been widely studied because of its extremely high application value. Taxadiene cyclase effectively converts GGPP to taxadiene, which is then hydroxylated by a series of P450s. Up to now, the complicated consecutive oxidation process catalyzed by various P450s has not been fully resolved. The first step is generally adapted to be the hydroxylation of the C-5 position of taxadiene by CYP725A4 to form taxadiene-5α-ol. The heterologous expression of CYP725A4 in *E. coli* and *S. cerevisiae* has found a variety of oxidation products along with taxadiene-5α-ol. It has been proposed that CYP725A2 and CYP725A1 then hydroxylate the C-13 and C-10 positions of taxadiene-5α-ol, respectively (Jennewein et al. 2001; Schoendorf et al. 2001). Furthermore, CYP725A5 and CYP725A6 catalyze the hydroxylation at the C-2 and C-7 sites of the alternative substrate taxusin (Chau and Croteau 2004; Chau et al. 2004). Further research discovered that CYP725A3 was able to introduce the hydroxy group at C-14, but it was not effective for the hydroxylation at the C-14 site in taxol (Jennewein et al. 2003) (Fig. 13). An enzyme of CYP716B subfamily from *Ginkgo biloba* (*G. biloba*) can hydroxylase the C-9 of taxane skeleton (Zhang et al. 2014).

**Fig. 14:**
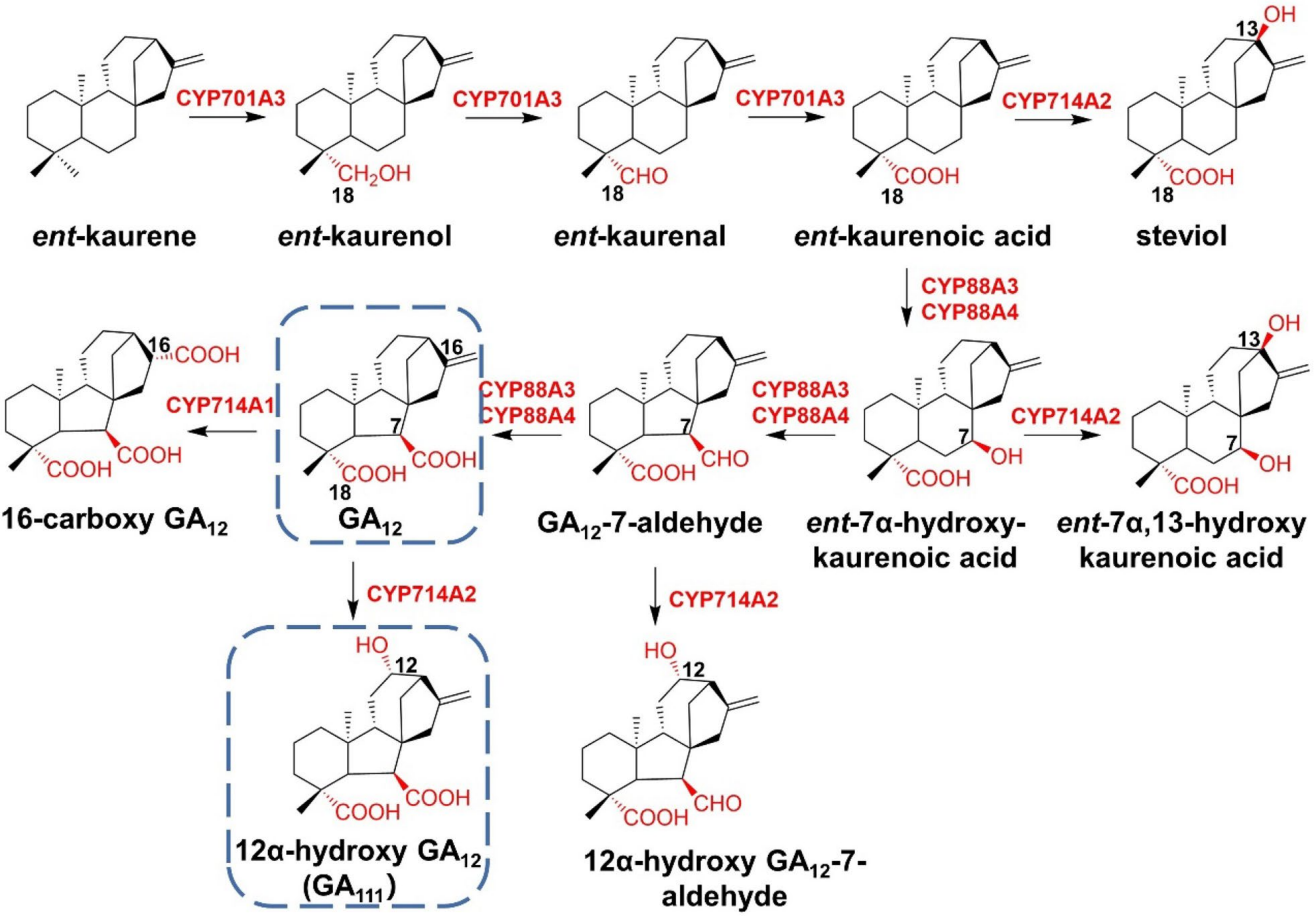
Proposed partial biosynthetic pathway of gibberellins, including GA_12_ and GA_111_

Gibberellins (GAs) is an essential plant hormone that can regulate plant growth and development, such as germination, flowering, and reproduction. The simplest gibberellin GA_12_ is assembled by diterpene synthase (diTPS) and P450s in a tandem manner. The diterpene synthase catalyzes the formation of *ent*-kaurene from GGPP, followed by a sequential three-step oxidative modification by the P450 subfamily, CYP701A and CYP88A. CYP701A3 from *A. thaliana* acts as an *ent*-kaurene oxidase to catalyze the three-step continuous oxidation of the C-18 position of *ent*-kaurene to produce *ent*-kaurenoic acid. After that, CYP88A3 and CYP88A4 perform the C-7 oxidation of *ent*-kaurenoic acid to deliver GA_12_ in three consecutive oxidation steps (Helliwell et al. 1999, 2001). Also extracted from *A. thaliana*, CYP714A1 protein has GA_12_ 16-carboxylation activity, while CYP714A2 can hydroxylate the C-13 or C-12 position of a variety of substrates in the metabolic pathway (Nomura et al. 2013) (Fig. 14).

### Triterpene hydroxylases

**Fig. 15:**
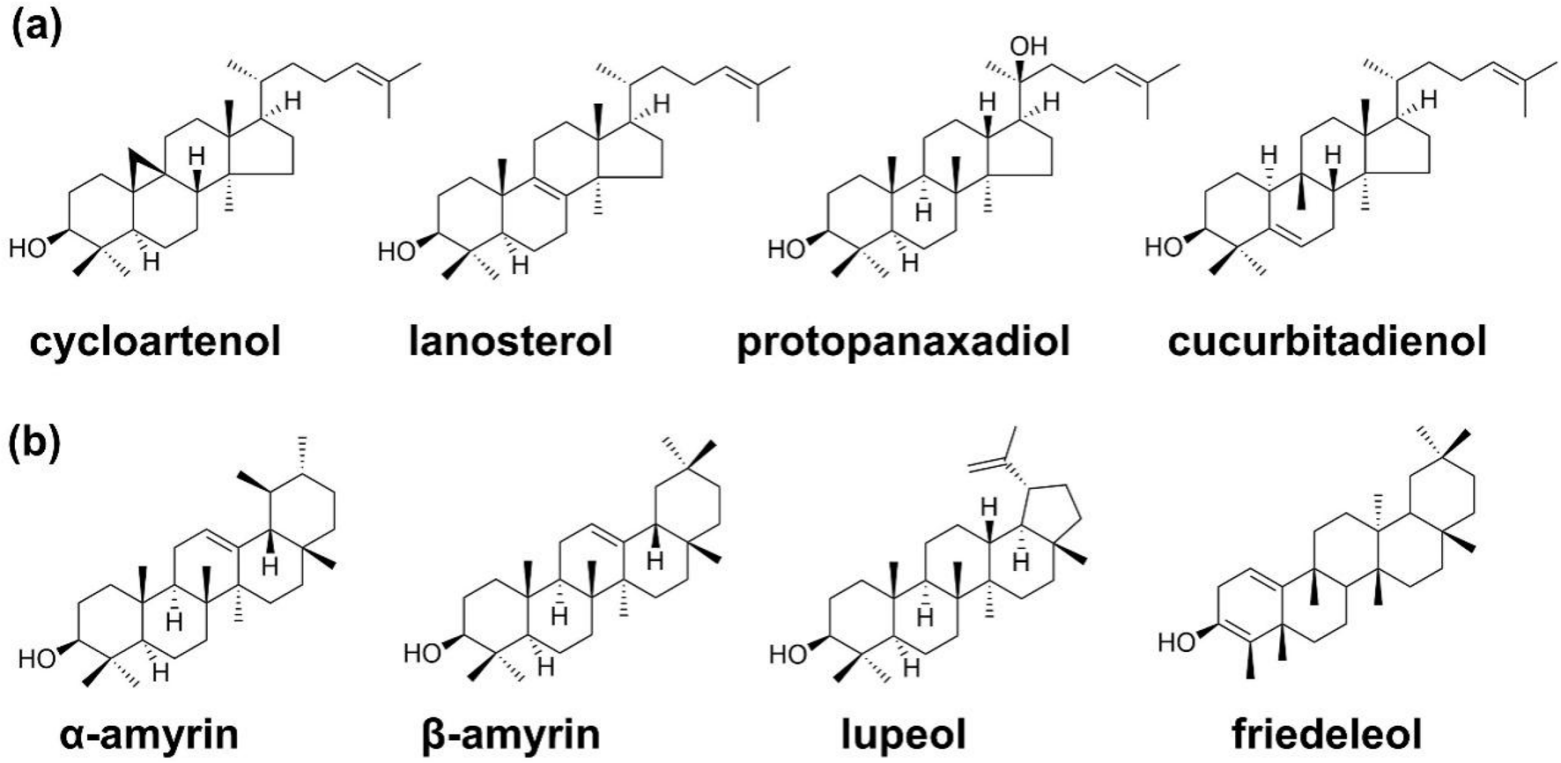
Triterpene skeletons derived from protosterol cation (**a**) and dammarenyl (**b**) subfamily

Triterpenoids are higher terpenoids containing six isoprene units and most of which exist in plant resins as free states or glycosides and esters. A few triterpenoids are also found in animals. Triterpene saponins are a class of metabolites with diverse structures and biological activities from a variety of plants. 2,3-Epoxy-squalene is the common precursor to all triterpenes, which are synthesized through the cyclization by oxidosqualene cyclase (OSC). It is generally believed that the triterpene cyclization stage will form two kinds of carbocations, protosterol cation and dammarenyl cation subfamily. Protosterol cations were used as the intermediates to form tetracyclic triterpenoids, such as cycloartenol, lanosterol, protopanaxadiol, and cucurbitadienol, while the pentacyclic triterpene skeletons, like α-amyrin, β-amyrin, lupeol, and friedeleol, were derived from the dammarenyl cations (Fig. 15). Skeleton modifications catalyzed by P450s and UDP-dependent glycosylation generate a variety of sapogenins with diverse structures. The oxidation by P450s contributes a lot to these structural variants and those P450s are mainly categorized as the CYP93, CYP88, CYP716, and CYP72 families.

**Fig. 16:**
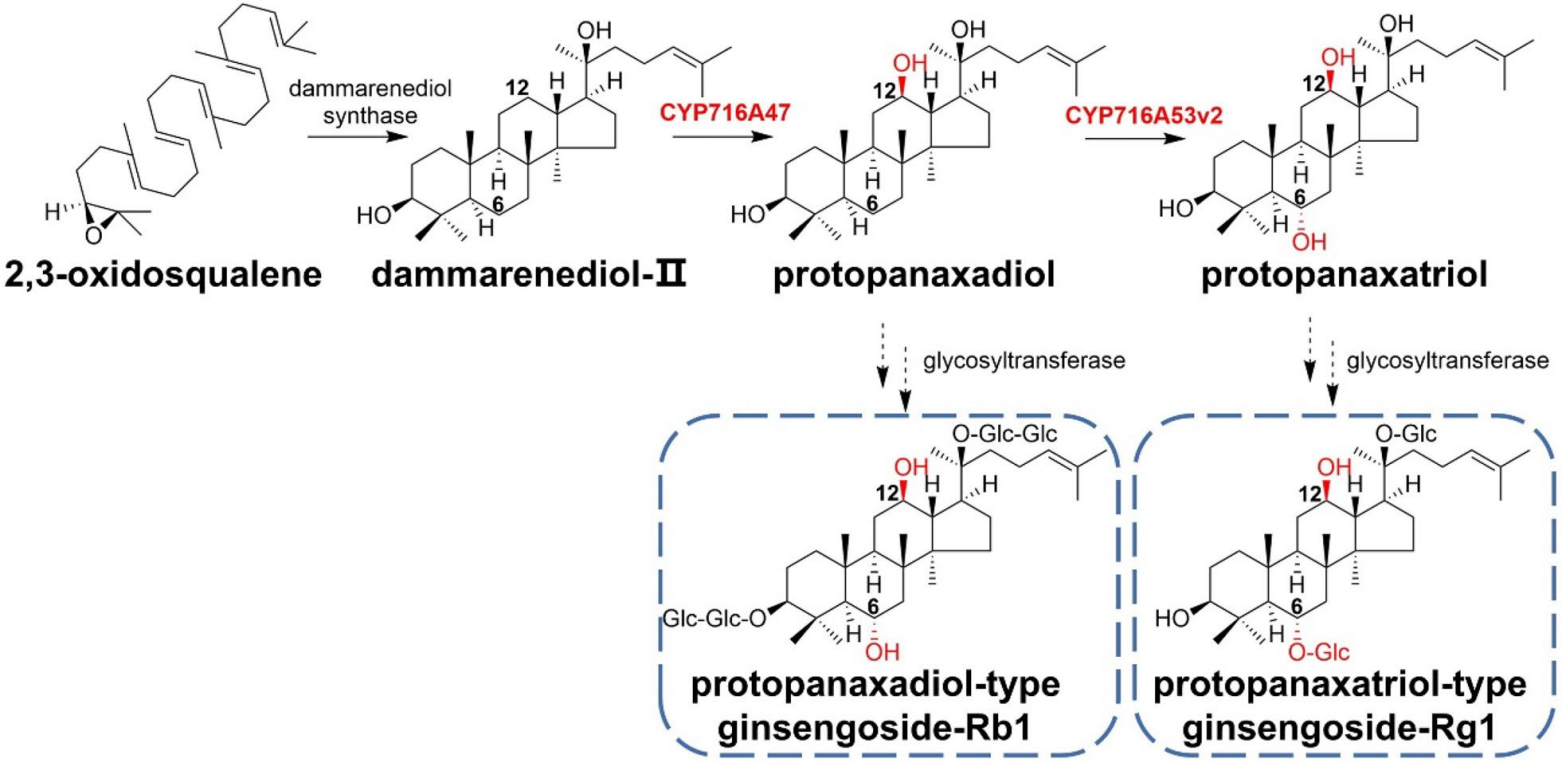
Proposed biosynthetic pathway for dammarenediol-type ginsenosides in *P. ginseng*

Ginseng is one of the most popular traditional medicinal materials and clinically display anti-stress, anticancer, anti-diabetic activities, and immune system modulation. The root contains the pharmacologically active ingredient ginsenosides derived from cytochrome P450 enzyme-catalyzed hydroxylation of the dammarenediol-II backbone and subsequent glycosyltransferase-catalyzed glycosylation. Han et al. successfully identified CYP716A47 as a protopanaxadiol synthase, which catalyzes the hydroxylation of the C-12 position of dammarenediol-II (Han et al. 2011). Subsequently, this team also isolated protopanaxadiol-6-hydroxylase CYP716A53v2 from *Panax ginseng* (*P. ginseng*) as a vital enzyme in ginsenoside biosynthesis (Han et al. 2012). CYP716A47 and CYP716A53v2 have strict substrate specificity (Fig. 16).

Mogroside is the main biologically active component extracted from *Momordica grosvenori* (*M. grosvenori*), which belongs to the cucurbitane-type tetracyclic triterpene saponins and is widely used as a natural sweetener worldwide. Mogroside is biosynthesized by a series of oxidations at the positions of C-11, C-24, and C-25 in the cucurbitadienol skeleton and subsequent glycosylations. The multifunctional enzyme CYP87D18 derived from *Siraitia grosvenorii* (*S. grosvenorii*) can carry out the C11-hydroxylation of cucurbitadienol to result in 11-hydroxycucurbitadienol, 11-oxo-cucurbitadienol, and 11-oxo-24,25-epoxy-cucurbitadienol (Zhang et al. 2016).

**Fig. 17:**
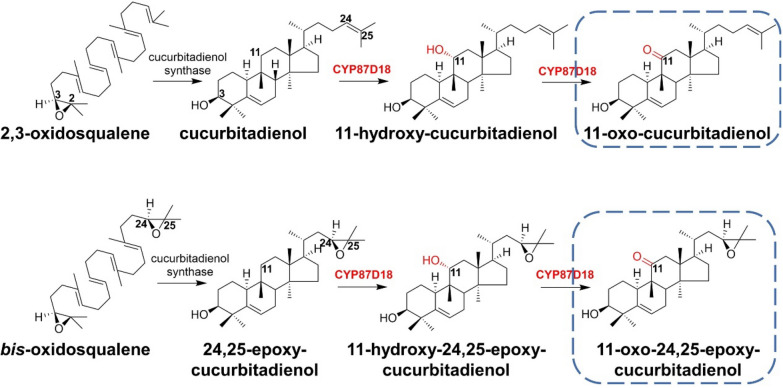
Proposed biosynthetic pathway for 11-oxo-cucurbitadienol and 11-oxo-24,25-epoxy-cucurbitadienol catalyzed by CYP87D18

Many studies have revealed that P450s are clustered together with the upstream biosynthetic gene in specific terpene biosynthetic pathways. For example, 19-hydroxycucurbitadienol-25-oxidase CYP81Q58 and cucurbitadienol-19-hydroxylase CYP88L2 were found in *Cucurbitaceae sativus* (*C. sativus*) to cluster together with cucurbitadienol synthase and other P450s, providing insights for the gene mining of new P450s (Shang et al. 2014) (Fig. 17).

**Fig. 18:**
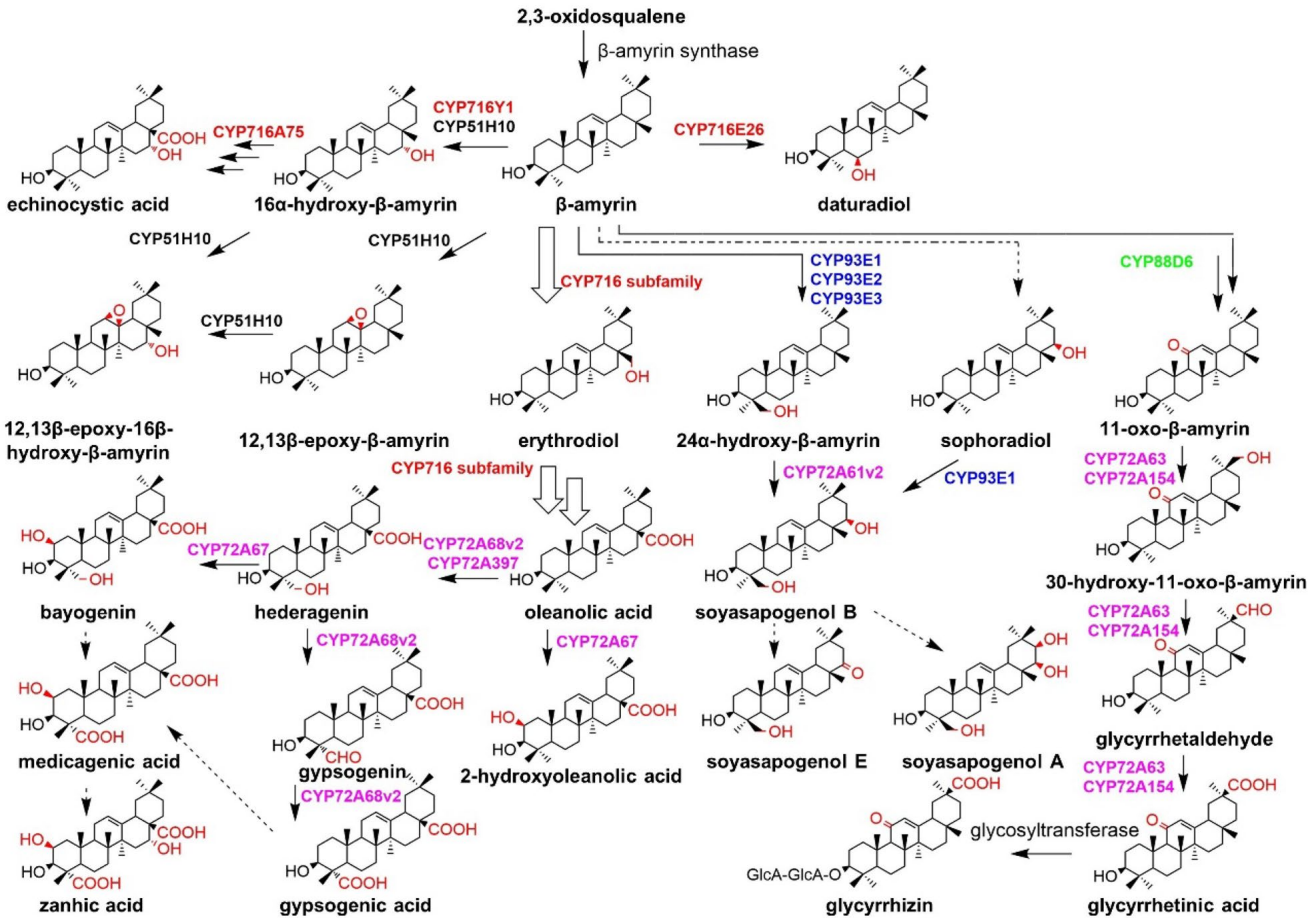
Biosynthesis pathways of β-amyrin skeleton triterpenoids. CYP716 subfamily in this figure: CYP716A1, CYP716A2, CYP716A12, CYP716A44, CYP716A46, CYP716A75, CYP716A94, CYP716AL1, CYP716A175, CYP716A179, CYP716A253, CYP716A52v2

β-Amyrin is the precursor for the biosynthesis of a series of triterpenoids, and many P450s can hydroxylate the β-amyrin skeleton to produce various triterpenoids with pharmacological activities (Fig. 18).

Glycyrrhizin is a triterpene saponin used as a natural sweetener with pharmacological indications, such as hepatoprotective, anti-virus, and anti-allergic activities. Glycyrrhizin is biosynthesized through a series of oxidations at the C-11 and C-30 positions of β-amyrin. CYP88D6 from *Glycyrrhiza uralensis* (*G. uralensis*) catalyzes the two-step continuous hydroxylation at the C-11 position of β-amyrin to produce 11-oxo-β-amyrin (Seki et al. 2008); CYP72A154 catalyzes the three-step continuous oxidation at the C-30 position of 11-oxo-β-amyrin to form glycyrrhetinic acid (Seki et al. 2011). CYP72A63, which has a higher sequence identity with CYP72A154, can also catalyze the hydroxylation of β-amyrin at the C-30 position. In addition, Zhu et al. identified its C-30 catalytic activity toward 11-oxo-β-amyrin with a higher catalytic specificity compared with CYP72A154 (Zhu et al. 2018).

Fukushima et al. confirmed two CYP72A subfamily enzymes (CYP72A61v2 and CYP72A68v2) that are highly related to the expression of CYP93E2 and CYP716A12 in the biosynthetic pathways of hemolytic and non-hemolytic sapogenins. CYP72A61v2 catalyzes the hydroxylation at the C-22 position of 24-hydroxy-β-amyrin to produce soyasapogenol B. Moreover, CYP72A68v2 catalyzes the C-24 position by three-step continuous oxidation of oleanolic acid to release an antibacterial agent gypsogenic acid (Fukushima et al. 2013).

Shibuya et al. firstly identified a triterpene hydroxylase CYP93E1 from *Glycine max* (*G. max*) with a catalytic activity of hydroxylation at the C-24 position of β-amyrin and sophoradiol (Shibuya et al. 2006). CYP93E3 (Seki et al. 2008) and CYP93E2 identified from *Medicago truncatula* (*M. truncatula*) also have shown the β-amyrin 24-hydroxylase activity (Fukushima et al. 2011). Fukushima et al. found that CYP716A12 co-expressed with β-amyrin synthase can catalyze the hydroxylation of β-amyrin, α-amyrin, and lupeol at the C-28 position and promote the continuous oxidation to yield oleanolic acid, ursolic acid, and betulinic acid (Fukushima et al. 2011). CYP716A94 (Han et al. 2018), CYP716AL1 (Huang et al. 2012), CYP716A175 (Andre et al. 2016), CYP716A52v2 (Han et al. 2013), CYP716A179 (Tamura et al. 2017), CYP716A1, and CYP716A2 (Yasumoto et al. 2016) of the CYP716 family also have the same functions as CYP716A12, while CYP716A44 and CYP716A46 only catalyze the oxidation of β-amyrin and α-amyrin to the corresponding acids. CYP716E26 catalyzes the hydroxylation of β-amyrin at the C-6 position to generate a rare biologically active triterpene, daturadiol (Yasumoto et al. 2017). CYP716A75 and CYP716A253 only catalyze the continuous oxidation of the C-28 position of β-amyrin to oleanolic acid, while CYP716A252 catalyzes the continuous oxidation of the C-28 position of α-amyrin to ursolic acid (Misra et al. 2017). CYP716A75 was found to promote the three-step continuous hydroxylation of the C-28 position of 16α-hydroxy-β-amyrin to the precursor 16α-hydroxy-oleanolic acid for maesa saponins. CYP716Y1 identified from *Bupleurum falcatum (B. falcatum)* can realize the hydroxylation of α-amyrin and β-amyrin at the C-16 position (Moses et al. 2014). Alternatively, CYP72A397 carries out the C-23 hydroxylation of oleanolic acid to give hederagenin with intriguing pharmacological properties (Han et al. 2018).

Tzin et al. reported other two CYP72A subfamily enzymes. CYP72A67 was found to catalyze the hydroxylation at the C-2 position of oleanolic acid and hederagenin. CYP72A68 catalyzes the three-step continuous oxidation of oleanolic acid to afford an antibacterial agent gypsogenic acid (Tzin et al. 2019). At present, a variety of P450 enzymes using β-amyrin as substrate have been identified in dicots, but only CYP51H10 has been discovered in monocots (Qi et al. 2006). CYP51H10 from *Avena strigosa* (*A. strigosa*) is a multifunctional P450 that can catalyze the oxidation of the C ring and D ring of β-amyrin to furnish the important precursor 12,13-β-epoxy-16β-hydroxy-β-amyrin for avenacins (Geisler et al. 2013).

### Tetraterpene hydroxylases

Tetraterpenoids feature a complex structure composed of eight isoprene units, of which carotenoids are the most representative tetraterpene pigments that are widely found in photosynthetic bacteria, some archaea, fungi, plants, and animals. As a photoprotective agent, antioxidant, and precursor of plant hormones, carotenoids are widely present in plants. Animals cannot synthesize carotenoids de novo, so those found in animals are usually ingested from plants and partly modified through metabolic reactions, rendering the structural diversity of animal carotenoids.

**Fig. 19:**
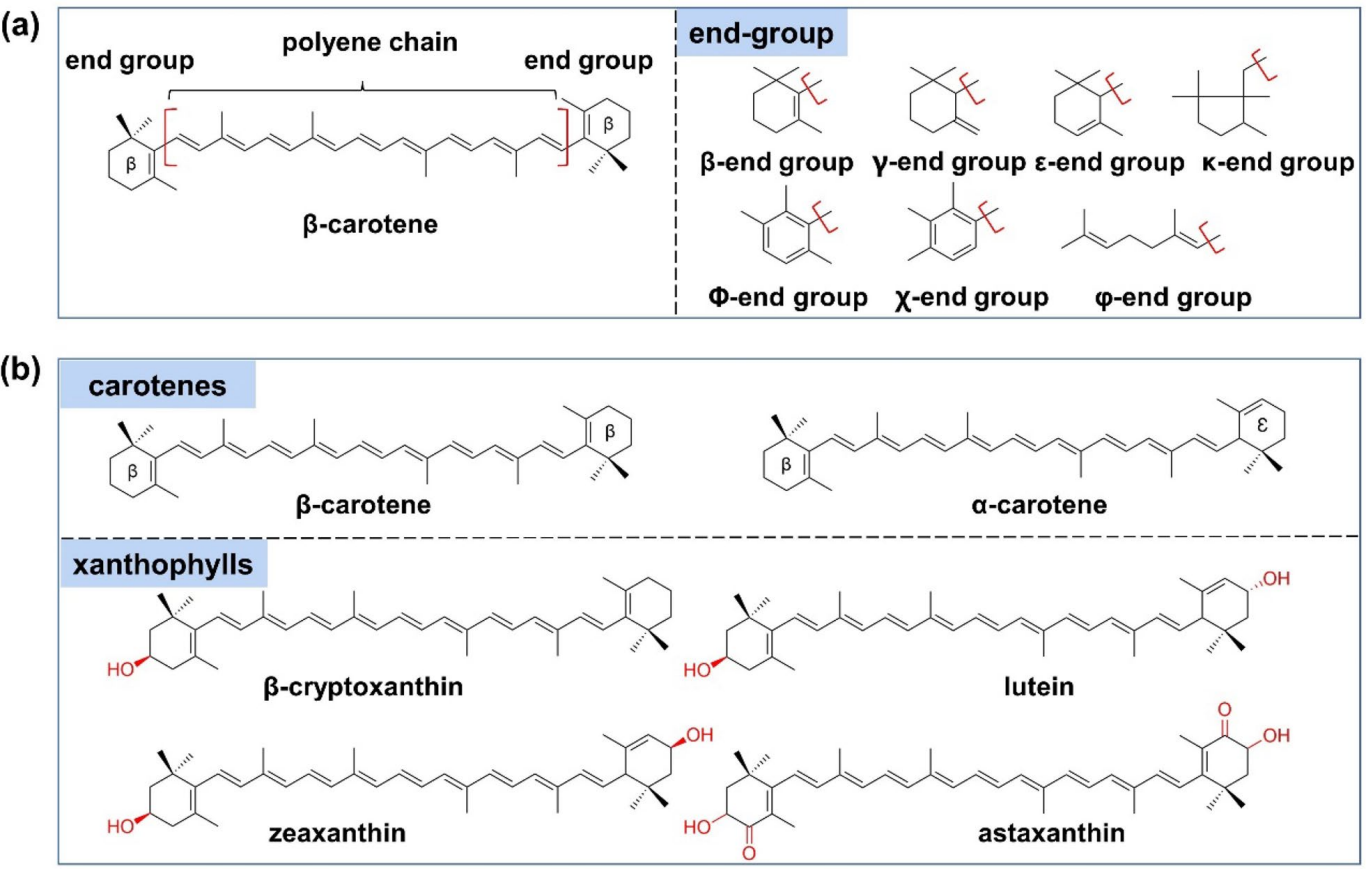
**a** Basic structures of carotenoids and end groups; **b** structures of typical carotenes and xanthophylls

There exist two kinds of carotenoids: carotenes, composed of hydrocarbons, including α-carotene, β-carotene, and γ-carotene; the other is xanthophylls containing several oxygen atoms which are introduced by various hydroxylases, such as hydroxyl, carboxyl, aldehyde, and epoxidation (Maoka 2020) (Fig. 19).

Two classes of structurally unrelated hydroxylases that can modify the rings of carotenes to xanthophylls, one is the heme-containing P450s and the other is the non-heme di-iron hydroxylases. CYP175A1, derived from the thermostable bacterium *Thermus thermophilus* HB27, shows a β-ring hydroxylase activity in the heterologous host *E. coli*, which was identified as the first β-carotene hydroxylase in the P450 superfamily.

**Fig. 20:**
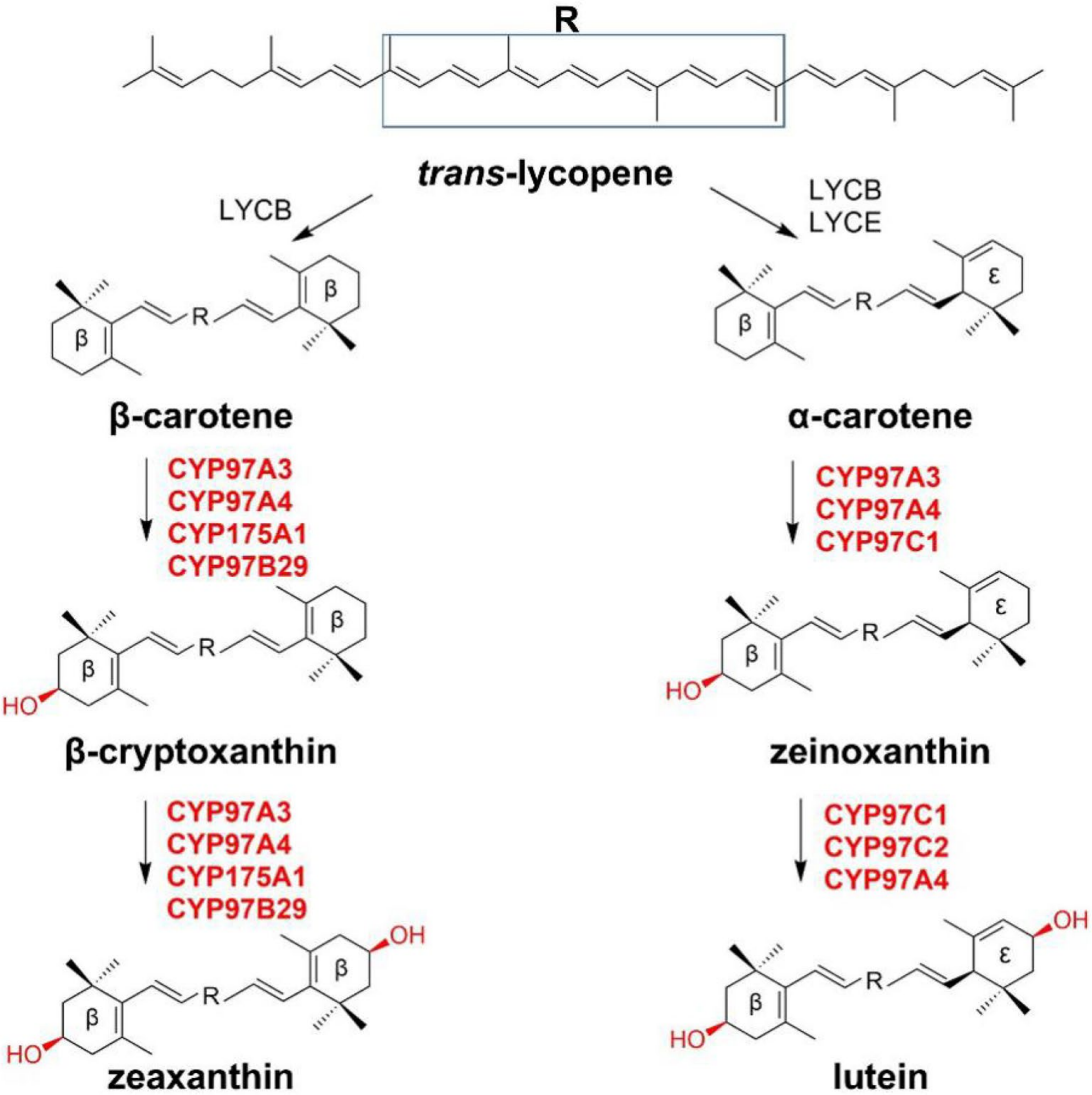
Biosynthetic pathway of Xanthophylls catalyzed by P450‐type carotene hydroxylases. *LYCB* lycopene β-cyclase, *LYCE* lycopene ε-cyclase

So far, all the cytochrome P450‐type carotene hydroxylases identified from plants belong to the CYP97 family. CYP97A3 and CYP97C1 from the phenotypes of the knock-out mutants of *A. thaliana* have exhibited activities against carotenoids. CYP97A3 can hydroxylate the two β-rings of β-carotene to produce zeaxanthin (Kim and DellaPenna 2006), while CYP97C1 can hydroxylate the β-ring and ε-ring to produce lutein (Tian and DellaPenna 2004). CYP97A4 and CYP97C2 from *Oryza sativa* have similar functions like the above two enzymes. CYP97A4 mainly acts as a β-ring carotene hydroxylase with minor activity toward the ε-rings, while CYP97C2 specifies the conversion of the ε-rings. Combined with the distribution of these hydroxylases and the accumulation of metabolites in plants, it is considered that Clan A enzymes in the CYP97 family are putative β-rings carotene hydroxylases, while Clan C enzymes are potential ε-rings carotene hydroxylases (Quinlan et al. 2007). Members of the CYP97B subfamily are more distant from Clans A and C with a wider distribution in plants. The first carotene β-rings hydroxylase CYP97B29 identified in *Red algae* can hydroxylate β-carotene to yield zeaxanthin (β,β-carotene-3,3′-diol). This hydroxylation reaction involves the extraction of hydrogen and the hydroxylation of the C-3 positions of the two β-rings (Yang et al. 2014). In addition, CYP97B3 was also found to be associated with carotenoid metabolism in *A. thaliana* (Kim et al. 2010) (Fig. 20).

Two non-heme di-iron hydroxylases CHXB (β-ring carotene hydroxylase) and CHXE (ε-ring carotene hydroxylase) from *Lycium chinense* (*L. chinense*) can hydroxylate α-carotene and β-carotene to form lutein (Zhao et al. 2014). In addition, a series of non-heme carotenoid hydroxylases from *A. thaliana*, green algae, and other plants, as well as from photosynthetic and non-photosynthetic bacteria have been cloned and functionally characterized in heterologous hosts (Galpaz et al. 2006; Kim et al. 2009; Linden 1999; Qi et al. 2020; Tian and DellaPenna 2004).

## The phylogenetic relationships of terpene hydroxylases

CYP450s involved in this article and their corresponding functions are listed in Table 1. As described above, on one hand, hydroxylases from different families or even different species may be able to accept terpene skeleton of a similar structure. For example, taxane hydroxylases derived from *Taxus* sp. all belong to CYP725A subfamily; however, an enzyme belonging to CYP716B subfamily derived from *G. biloba* was found to be able to hydroxylate the C-9 position of the taxane skeleton. Similarly, enzymes of the CYP97 family are mainly involved in the biosynthesis of tetrapenoid zeaxanthin; however, CYP175A1 was also found to hydroxylate β-cryptoxanthin to produce zeaxanthin. On the other hand, the metabolic pathway of some structurally similar substrates often involves enzyme from several specific families. For example, the vast majority of biocatalysts capable of hydroxylating or oxidizing β-amyrin backbone-type triterpenes belong to CYP716, CYP88, and CYP72 (Fig. 18). It seems that some associations exist between the specific CYP450 family and their substrate spectrum.

**Table 1:** CYP450s mentioned in this article and brief descriptions of their functions

Classification	CYP450s	Functions
Monoterpene hydroxylases	CYP76C1	Linalool hydroxylase
	CYP76C2	Citronellol 8-hydroxylase
	CYP76C4	Linalool hydroxylase
	CYP76B6	Nerol 8-hydroxylase
	CYP71B31	Linalool hydroxylase
	CYP76C3	Linalool hydroxylase
	CYP76F45	Geraniol 8-hydroxylase
	CYP6BH5	Geraniol 8-hydroxylase
	CYP153A6	Limonene 7-hydroxylase
	CYP750B1	(+)-Sabinene synthase
	CYP76AA25	Sabinene-hydroxylase
	CYP345E2	Limonene-hydroxylase
	CYP6DE1	Pinene-hydroxylase
	CYP6DE3	Pinene-hydroxylase
	CYP6DJ1	Limonene-hydroxylase
	CYP71D12	Tabersonine hydroxylase
	CYP71D351	Tabersonine hydroxylase
	CYP71BJ1	Tabersonine hydroxylase
Sesquiterpene hydroxylases	CYP71AV2	GA oxidase
	CYP71BL2	GAA 6-hydroxylase
	CYP71BL1	GAA 8-hydroxylase
	CYP71AV9	GA hydroxylase
	CYP71BL5	Costunolide synthase
	CYP260A1	Unspecific monooxygenase
	CYP264B1	Unspecific monooxygenase
	CYP71AV1	Amorpha-4,11-diene monooxygenase
	CYP71AV8	(+)-Valencene hydroxylase
	CYP109B1	Valencene oxidase
	CYP71BE5	α-Guaiene hydroxylase
	CYP71D51V2	(+)-Valencene 2-hydroxylase
	CYP183A1	Pentalenene 13-hydroxylase
	CYP170A1	*epi*-Isozizaene monooxygenase
Diterpene hydroxylases	CYP71D16	CBT-ol 6-hydroxylase
	CYP102A1 F87A/I263L	*β*-CBT-diol 9-hydroxylase
	CYP102A1 L75A/V78A/F87G	*β*-CBT-triol 10-hydroxylase
	CYP726A14	Casbene 5-oxidase
	CYP726A17	Casbene 5-oxidase
	CYP726A18	Casbene 5-oxidase
	CYP726A16	5-Keto-casbene 7,8-epoxylase
	CYP726A15	Neocembrene 5-oxidase
	CYP726A19	Casbene 5-oxidase
	CYP71D445	Casbene hydroxylase
	CYP726A27	Casbene hydroxylase
	CYP76AH1	Ferruginol synthase, ferruginol 11-hydroxylase
	CYP76AH4	Ferruginol synthase, ferruginol 11-hydroxylase
	CYP76AH5	Ferruginol synthase, ferruginol 11-hydroxylase
	CYP76AH6	Ferruginol synthase, ferruginol 11-hydroxylase
	CYP76AH7	Ferruginol synthase, ferruginol 11-hydroxylase
	CYP76AH22	Ferruginol synthase, ferruginol 11-hydroxylase
	CYP76AH23	Ferruginol synthase, ferruginol 11-hydroxylase
	CYP76AH24	Ferruginol synthase, ferruginol 11-hydroxylase
	CYP76AK6	11-Hydroxyferruginol 20-oxidase, miltiradiene 20-hydroxylase
	CYP76AK7	11-Hydroxyferruginol 20-oxidase, miltiradiene 20-hydroxylase
	CYP76AK8	11-Hydroxyferruginol 20-oxidase, miltiradiene 20-hydroxylase
	CYP76AH3	Ferruginol oxidase
	CYP76AK1	11-Hydroxyferruginol 20-oxidase
	CYP720B1	Resin acids 18-oxidase
	CYP720B4	Resin acids 18-oxidase
	CYP725A1	Taxane 10-hydroxylase
	CYP725A2	Taxane 13-hydroxylase
	CYP725A3	Taxane 14-hydroxylase
	CYP725A4	Taxadiene 5-monoxygenase
	CYP725A5	taxane 2-hydroxylase
	CYP725A6	Taxane 7-hydroxylase
	CYP701A3	*ent*-Kaurene 18-oxidase
	CYP88A3	*ent*-Kaurenoic acid 7-oxidase
	CYP88A4	*ent*-Kaurenoic acid 7-oxidase
	CYP714A1	GA_12_ 16-carboxidase
	CYP714A2	Gas hydroxylase
Triterpene hydroxylases	CYP716A47	Dammarenediol-II 12-hydroxylase
	CYP716A53v2	Protopanaxadiol 6-hydroxylase
	CYP87D18	Cucurbitadienol 11-hydroxylase
	CYP81Q58	19-Hydroxycucurbitadienol-25-oxidase
	CYP88L2	Cucurbitadienol-19-hydroxylase
	CYP88D6	β-Amyrin 11-oxidase
	CYP72A154	11-Oxo-β-amyrin 30-oxidase
	CYP72A63	β-Amyrin 30-hydroxylase
	CYP72A61v2	24-Hydroxy-β-amyrin 22-hydroxylase
	CYP72A68v2	Oleanolic acid 24-oxidase
	CYP93E2	β-Amyrin 24-hydroxylase
	CYP93E3	β-Amyrin 24-hydroxylase
	CYP716A12	β-Amyrin 28-hydroxylase
	CYP93E1	β-Amyrin 24-hydroxylase
	CYP716A94	β-Amyrin 28-hydroxylase
	CYP716AL1	β-Amyrin 28-hydroxylase
	CYP716A175	β-Amyrin 28-hydroxylase
	CYP716A52v2	β-Amyrin 28-hydroxylase
	CYP716A179	β-Amyrin 28-hydroxylase
	CYP716A1	β-Amyrin 28-hydroxylase
	CYP716A2	β-Amyrin 28-hydroxylase
	CYP716A44	β-Amyrin oxidase, α-amyrin oxidase
	CYP716A46	β-Amyrin oxidase, α-amyrin oxidase
	CYP716E26	β-Amyrin 6-hydroxylase
	CYP716A75	β-Amyrin 28-oxidase, 16α-hydroxy-β-amyrin 28-oxidase
	CYP716A253	β-Amyrin 28-oxidase
	CYP716A252	α-Amyrin 28-oxidase
	CYP716Y1	β-Amyrin 16-hydroxylase, α-amyrin 16-hydroxylase
	CYP72A397	Oleanolic acid 23-hydroxylase
	CYP72A67	Oleanolic acid 2-hydroxylase, hederagenin 2-hydroxylase
	CYP72A68	Oleanolic acid oxidase
	CYP51H10	β-Amyrin oxidase
Tetraterpene hydroxylases	CYP175A1	β-Carotene β-ring hydroxylase
	CYP97A3	β-Carotene β-ring hydroxylase
	CYP97C1	β-Carotene β-ring hydroxylase, β-carotene ε-ring hydroxylase
	CYP97A4	β-Carotene β-ring hydroxylase
	CYP97C2	Zeinoxanthin ε-ring hydroxylase
	CYP97B29	β-Carotene β-ring hydroxylase
	CYP97B3	Carotenoid oxidase

**Fig. 21:**
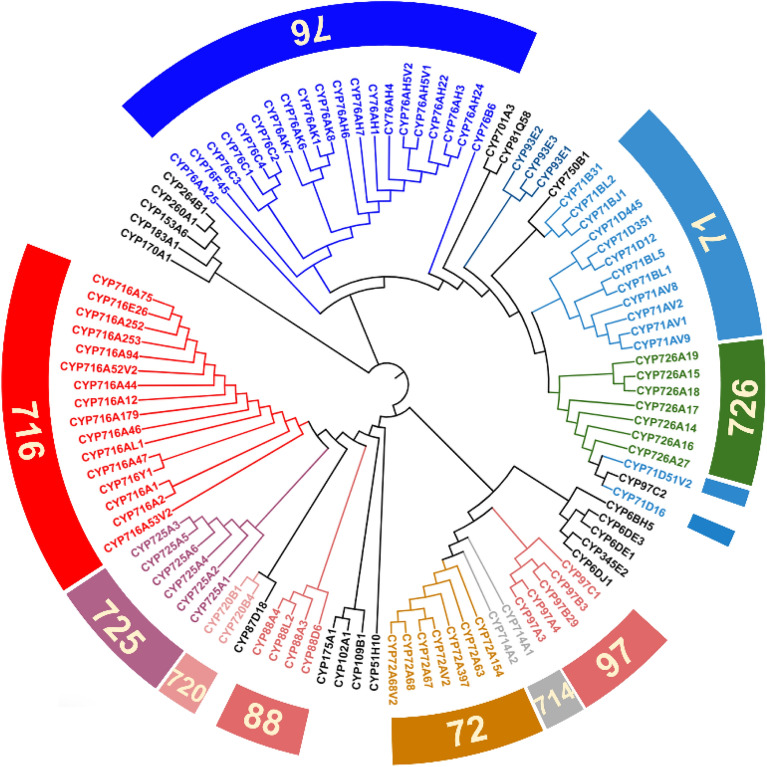
The phylogenetic tree of CYP450s involved in this article

We established the phylogenetic relationship of a total of 108 terpene hydroxylases involved in this paper, with the purpose to explore the connections between these different hydroxylases from the perspective of evolution (Fig. 21). In general, CYP76 and CYP71 families are involved in the biosynthesis of monoterpenes (Fig. 2); CYP71, CYP264, and CYP260 families are involved in the biosynthesis of sesquiterpenes (Fig. 8); CYP726, CYP725, and CYP716 families are mainly involved in the biosynthesis of diterpenes; and CYP97 and CYP175 are involved in the biosynthesis of tetraterpene (Fig. 20). It is obvious that as the size of substrate backbone gradually increases, the location of the corresponding CYP450 families on the phylogenetic tree gradually transits from one side (feature with blue and green) to another side (feature with red and orange). Some individual exceptions exist, for example, CYP701A3 is able to hydroxylate the C-18 position of GA (Fig. 14), yet it is more closely related to the CYP71 and CYP76 families and more distant from the branch where other triterpene hydroxylases are located. However, individual examples did not hinder the overall phylogenetic trend.

Different CYP450s capable to accept similar substrates often have a close phylogenetic relationship. Besides, it is obvious from the evolutionary tree that biocatalysts of these three families (CYP76, CYP71, and CYP726) evolved from the same ancestral enzyme. As a result, it is presumed there exists a close evolutionary connection between monoterpenes hydroxylases and sesquiterpenes hydroxylases. As a contrast, the core skeletons of diterpenes, triterpenes, and tetrapenes are often rather large, and it seems that their corresponding hydroxylases are more evolved from another ancestral enzyme.

These phenomena provide some novel ideas for the digging of new terpene hydroxylases. When we need to search for hydroxylases with specific substrate backbones, it is feasible to mine and screen CYP450s from some specific families. For instance, it seems that there exists a higher probability of finding new monoterpene hydroxylases from CYP76, CYP71 families, or other families which have a closer phylogenetic relationship with these two CYP450 families.

## Optimize expression, activity, and selectivity of terpenoid hydroxylases

For a long time, terpenoids with intriguing physiological and pharmacological activities have emerged as the appealing topics in the community. From the extraction of active ingredients to the isolation and purification of single compound, more and more terpenoids have been characterized and investigated in the biological pathways.

CYP450s involved in the biosynthetic pathway of terpenoids remain a main bottleneck for clarifying the detailed function, retarding to display the full capacity of oxidases in producing significant substances. These P450s have problems, such as low expression, poor stability, as well as low activity. At the same time, the catalytic promiscuity, which means these hydroxylases can accept a variety of substrates or catalyze one substrate to generate a variety of products, is also a significant obstacle resulting in the dispersion of metabolic pathways and further reducing the yield of desired products. At present, there are many studies focusing on solving these problems, among which the heterologous expression of these hydroxylases has been significantly improved. Meanwhile, some progresses have been made aiming at the catalytic promiscuity and low activity of these hydroxylases. The challenges and corresponding solutions of these CYP450s are summarized in the following part.

### Optimize the functional expression of terpene hydroxylases

Synthetic biology techniques generally choose to construct metabolic pathways for the terpenoid production in easily cultured organisms, such as the bacterial system *E. coli* or the fungal system *S. cerevisiae*. Most terpene hydroxylases, especially those derived from eukaryotes, belong to type II P450s and bind to the endoplasmic reticulum membrane. *S. cerevisiae* are generally more suitable for the expression of CYP450s of eukaryotic origin because of their natural endosomal system. Optimizing promoter strength as well as increasing gene copy number are methods that are usually used to improve protein expression in *S. cerevisiae* (Walls et al. 2021). However, in general, the functional expression of these CYP450s in *E. coli* is challenging because of the inherent limitations of the bacterial platform. Firstly, *E. coli* lacks membrane structures, such as endoplasmic reticulum, which is incompatible with the translation of the membrane signaling modules of membrane-bound CYP450s. Secondly, the lack of CYP450 reductases (CPRs) in the prokaryotic system cannot transmit electrons for CYP, that hinders the catalytic function of CYP. Therefore, in general, membrane proteins, such as CYP725A4, are difficult to functionally express in bacterial systems.

A variety of measures have been developed to achieve the functional expression of these membrane-bound CYP450s in easy-to-handle model heterologous host *E. coli*, including *N-*terminal modification or co-expression of chaperones.

#### *N*-terminal modification

The *N*-terminus of type II P450s are often composed of hydrophobic amino acids which can bind to the endoplasmic reticulum and exert catalytic function. The predict protein software (http://www.predictprotein.org/) can be used to identify the transmembrane region of these membrane-bound P450s. This hydrophobic sequence always becomes an obstacle to heterologous expression in *E. coli*. As a result, part of the* N*-terminal hydrophobic sequence was often truncated to increase the solubility of the protein and promote its correct folding. On the contrary, some hydrophilic sequences can be artificially added in *N*-terminus.

The inclusion body of 5α-hydroxylase for taxadiene (CYP725A4 from *Taxus*) is obtained by directly expressing the enzyme in *E. coli*, and several groups have developed various modification strategies to promote its soluble expression. Rouck et al. obtained three constructs MA, 2b1, and 17α through different modifications to the* N*-terminal of CYP725A4 (Rouck et al. 2017)_._ MA herein represents that the *N*-terminal 59 amino acids of CYP725A4 have been truncated and Met-Ala was added to the starting position which can help increase the soluble expression; 2b1 is based on the truncation of 59 amino acids at the *N*-terminal and replaced with a peptide MAKKTSSKGKLPPGPS from the *N-*terminal of rabbit CYP2b1; 17α means that the *N*-terminal 24 amino acids of CYP725A4 are truncated and a sequence of MALLLAVF from bovine steroid hydroxylase is added. The yields of three purified proteins obtained in *E. coli* were 1.54 nmol/L, 1.76 nmol/L and 4.83 nmol/L, respectively, indicating that this *N*-terminal modification method has a certain positive effect on the soluble expression of P450s.

**Fig. 22:**
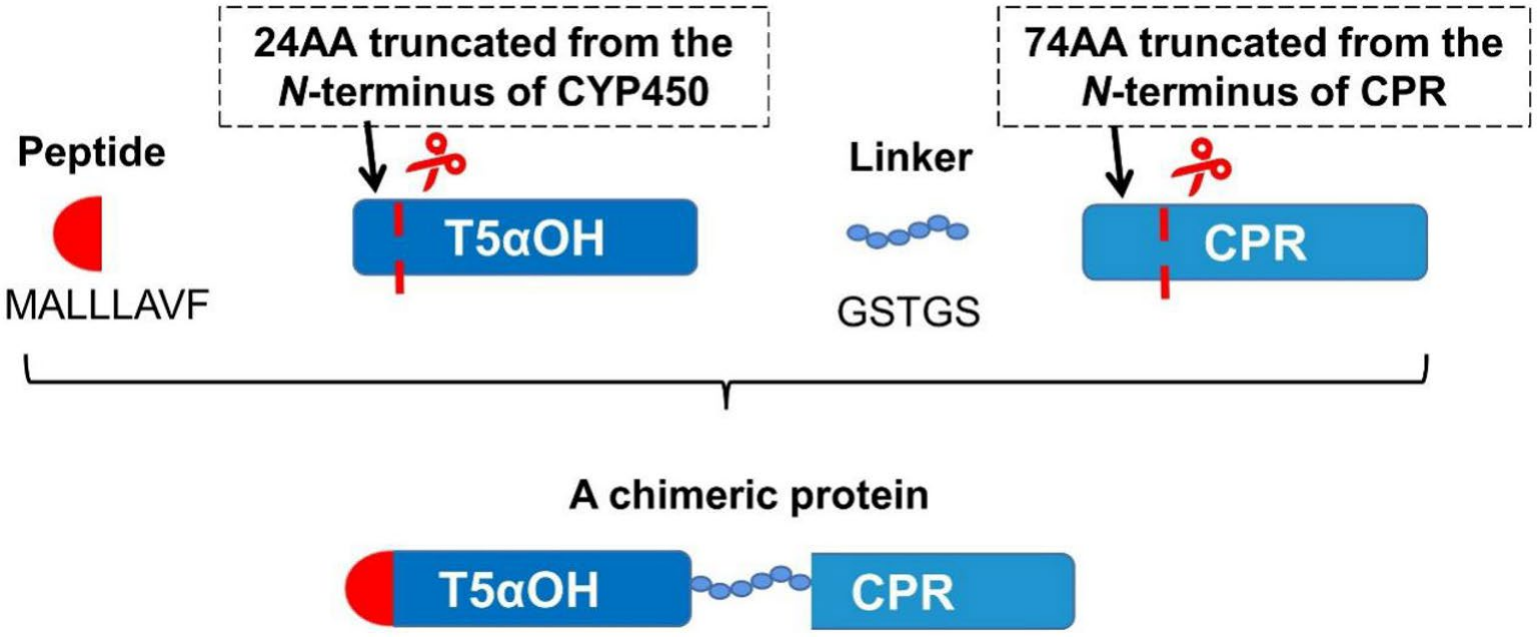
Schematic diagram of chimeric protein construction. At24T5αOH-tTCPR was taken as a typical example

Ajikumar et al. truncated the *N*-terminus of CYP725A4 and CPR with different lengths through transmembrane engineering by adding MALLLAVF into the *N*-terminus of CYP725A4. The two truncated proteins were connected through the GSTGS linker to construct a chimeric protein (Fig. 22). Among the constructed chimeric proteins, At24T5αOH-tTCPR has the highest conversion rate to the substrate taxadiene, of which CYP725A4 was truncated by 24 amino acids and *Taxus*-derived CPR truncated 74 amino acids in the *N*-terminus endoplasmic reticulum binding region (Ajikumar et al. 2010).

#### Co-expression of molecular chaperones

Some P450s co-expressed with molecular chaperones to provide a suitable hydrophilic environment to help the correct folding of P450s and the proper insertion of heme. Ichinose et al. used different modification methods to enable 304 P450s derived from fungi to be functionally expressed in *E. coli.* Among them, CYP505D8v1, CYP5137A4v1, and CYP505D6 use molecular chaperones GroES/GroEL to help proteins fold correctly, and their volume titers reached 1333 nmol/L, 1820 nmol/L, and 665 nmol/L, respectively. CYP5139D7v1, CYP5147B1, and CYP5150A2 were truncated to varying degrees of *N*-terminal, and the co-expression of GroES/GroEL also helped these P450s fold correctly. CYP5037E1 and CYP5149A1 were reconstructed by *N*-terminal modification and replaced with the *N*-terminal domain of CYP5144C1 to construct a chimeric protein to achieve a volume titer of 2000 nmol/L, which is currently the highest yield of fungal P450s expressed in *E. coli*. These reports have important references for expressing P450s from other eukaryotic sources in *E. coli* (Fatima et al. 2021; Ichinose et al. 2015).

### Improve the catalytic activity and chemo- or regioselectivity of terpene hydroxylases

#### Co-expression of external redox partner

**Fig. 23:**
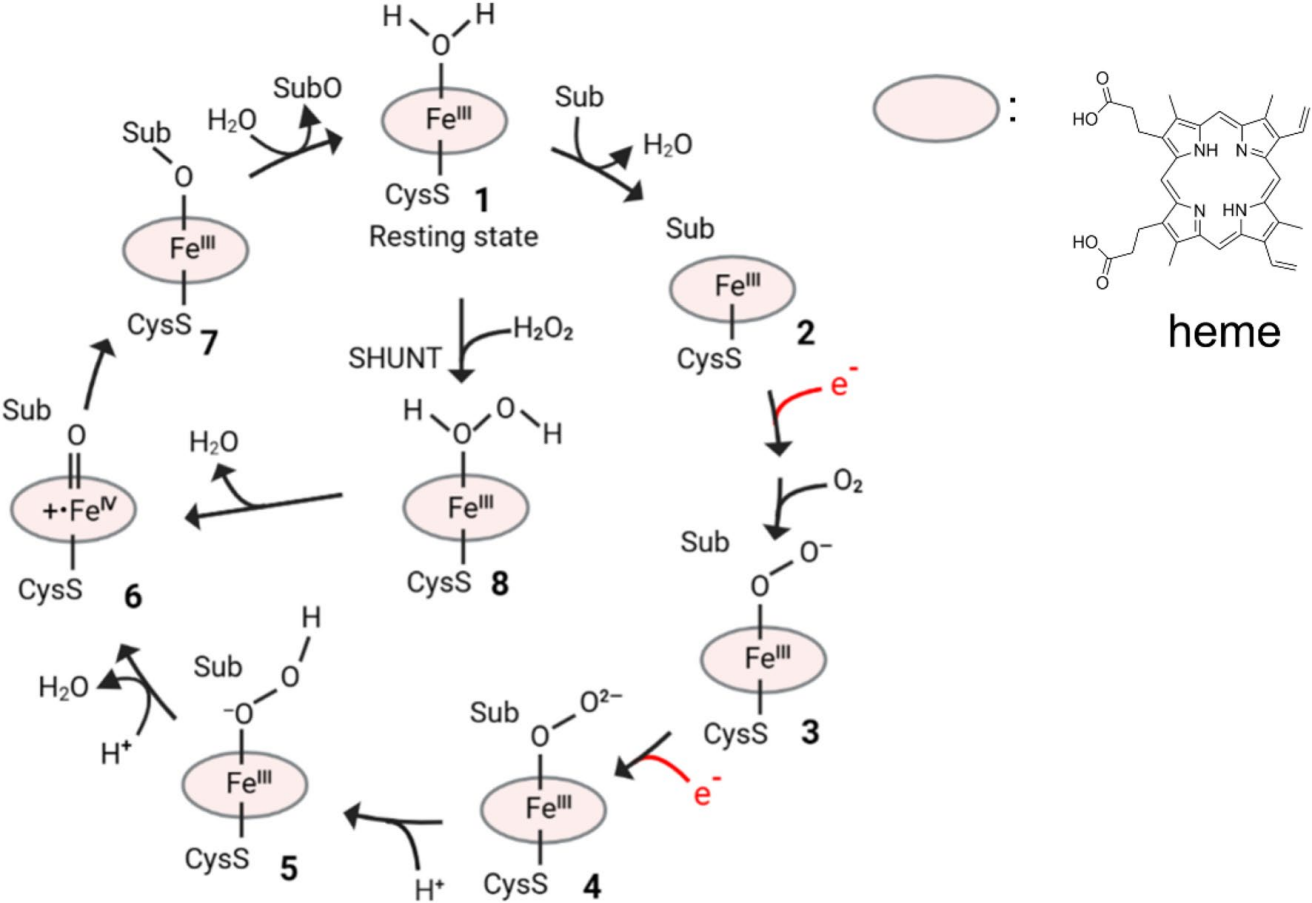
Typical catalytic cycle of P450s for hydroxylations. The exact structure of heme is marked on this diagram

Cytochrome P450 reductase (CPR), also known as NADPH-dependent cytochrome P450 oxidoreductase, is a membrane-bound oxidoreductase embedded in the cytosolic side of the endoplasmic reticulum. CPR is indispensable in most CYP450-catalyzed reactions, responsible for sequentially delivering two electrons from the cofactor NAD(P)H to the heme Fe. The first electron in CPR reduces the heme Fe and promotes the O_2_ binding to form a superoxide complex (**3**), and the second electron further reduces **3** to a peroxide complex (Fe^3+^OO)^2−^ P450 (**4**). The subsequent reaction oxidizes the substrate and produces a molecule of water (Fig. 23). CPR modulates the activities of P450s through altering the electron transfer processes. In addition, the protein–protein interaction (PPI) between chimera CYP450 and CPR may alter the spatial conformation of CYP450, thus affecting the substrate binding (Zhang et al. 2022; Sun et al. 2020).

In general, the electron transfer system expression hosts, such as *E. coli* and *S. cerevisiae,* are not completely matched to P450s originated from plants. Therefore, it is often necessary to co-express homologous CPR helping P450s to exert their physiological effects. At present, the most commonly used eukaryotic expression hosts *S. cerevisiae* WAT11 and WAT21 are constructed by using the CPRs from *A. thaliana*-derived ATR1 and ATR2 to replace the endogenous genes of CPR1 and CPR2, respectively (Pompon et al. 1996).

Zhu et al. screened six CPRs derived from different plants, including ATR1 and ATR2 from *A. thaliana*, MTR2 and MTR3 from *Medicago sativa*, LjCPR1 from *Lotus japonicus*, and GuCPR1 from *G. uralensis*. Their coupling efficiencies with CYP72A154, a CYP450 involved in the biosynthesis of glycyrrhetinic acid, were examined by inserting the genes of CYP72A154 and the corresponding CPR into the genome of engineered *S. cerevisiae* INVSc1. The production of GA and other related triterpenoids were varied significantly when different CPR was used, with the engineered strain containing GuCPR1 had the highest titer of GA. This phenomenon revealed that CPRs have the capability to affect the catalytic activity of CYP450 and further altered the overall productivity of terpenoids (Zhu et al. 2018).

Chang et al. showed when CYP706B1 (cadinene C-8 hydroxylase) was co-transformed into the engineered *E. coli* with aaCPRct from *Artemisia annua* with a 1.8-fold decrease in the overall productivity of 8-hydroxycadinene, compared with that co-transformed with ctCPR from *Candida tropicalis*. Since these two CPRs did not show apparent difference in the in vitro cytochrome *c* reduction activity, this phenomenon seems to be attributed to the protein–protein interactions between CYP450 and CPRs (Chang et al. 2007).

The low electron transfer efficiency between CPR and P450 is one of the main reasons rendering the low catalytic activity of P450s. Very recently, Park et al. connected CPR and P450 with the self-assembled scaffold protein CipB from *Photorhabdus luminescens*, respectively. After connected with this scaffold protein, CPR and P450 can spontaneously form the protein crystal inclusion (PCI) in *E. coli*, which shortened the distance between CPR and P450, as a result increasing the efficiency of CPR transferring electrons to P450. This strategy was further applied to the P450s related to the biosynthesis pathway of lutein, (+)-nootkatone, apigenin, and l-3,4-dihydroxyphenylalanine (l-DOPA), respectively. The production of these compounds in engineered *E. coli* was significantly increased, proving that using scaffold proteins to assist electron transfer between CPRs and P450s was capable for universally application (Park et al. 2022).

Additionally, a single CPR can supply electrons to multiple P450s in natural product biosynthetic pathways. It was founded that the ratio of CPR and CYP450s achieve 1:15 in the microsomal membrane (Jensen and Møller 2010). To minimize the generation of toxic reactive oxygen species caused by the disruption of the degree of coupling between the CPR and CYP450s, Walls et al. expressed CYP725A4 under GAL1 promoter to maximize the expression of CYP450, while expressed CPR under the weak GAL3 promoter in *S. cerevisiae* (Walls et al. 2021).

#### Molecular modification of terpene hydroxylases

Eukaryotic terpenoid hydroxylases, especially those derived from plants, are typically poorly expressed in heterologous hosts with low stability. These membrane proteins are difficult to be purified or crystallized, and as a result, structural information about these enzymes are extremely rare. On the other hand, lacking of high-throughput screening methods render traditional directed evolution methods, such as error-prone PCR, which require the screening of a large number of mutant libraries, to be difficult to apply in the modification of terpene hydroxylases.

Much of what is known about CYP450 structure–function relationships and molecular modification of the activity and selectivity of terpene hydroxylases is therefore based on the homologous models of the structure of CYP450s already clarified from bacterium. Small mutant libraries are designed toward the active centers or a specific secondary structure area of those hydroxylases, and the viability, regional selectivity, and stereoselectivity of mutants were further characterized by intracellular fermentation experiments. Additionally, hydroxylases capable of hydroxylating different locations of the same terpene backbone often originate from a same CYP450 subfamily with high sequence identity among each other. Therefore, analysis of the evolutionary data of amino acid is also a feasible method to locate the key amino acids that affect the selectivity or activity of terpenoid hydroxylases.

1. **Manipulate the regioselectivity of terpenoid hydroxylases**
  Limonene-3-hydroxylases CYP71D13 and CYP71D15 and limonene-6-hydroxylase CYP71D18 have been identified from mint oil gland cDNA libraries. CYP71D13 and CYP71D15 were found to exhibit 93% sequence identity with each other and showed around 70% sequence identity with CYP71D18. The exploration of regioselectivity of limonene hydroxylases mainly started from the sequence alignment results. Firstly, through building chimeric enzymes to exchange large restriction fragments and exchange of subdomains of CYP71D15 and CYP71D18, a subdomain with a total length around 20 amino acids is localized, which had a direct effect on the hydroxylation regiospecificity of the product. It was found that only 5 amino acids were different in this subdomain, and therefore, reciprocal site-directed mutagenesis was designed for these five mutation sites. Interestingly, after changing Phe363 of CYP71D18 to Ile, the selectivity of its hydroxylation position was reversed from the C-6 to C-3 position, indicating that the residues at site 363 play an important role in the binding of the substrate and affected the regiospecific preference (Schalk and Croteau 2000).
  Moreover, Mau et al. identified CYP71D174 from *Perilla frutescens* as limonene-7-hydroxylase by using the cDNA sequence of limonene-3-hydroxylase and limonene-6-hydroxylase as probes. CYP71D174 is 63% and about 69% identical with CYP71D18 and CYP71D13, respectively. The amino acid of Phe at position 363 of CYP71D174 catalyzes (−)-limonene to produce the C-7, C-3, and C-6 oxygenated products. This phenomenon showed that besides the amino acid at position 363, there exist other amino acids involved in the immobilization of the substrate in the active center (Mau et al. 2010).
2. **Expanding the substrate spectrum and modify substrate heterogeneity of terpenoid hydroxylases**
  In the biosynthesis of cucumber cucurbitacin C (CuC), CYP87D20 from *Cucumis sativus* L. catalyzes the C11-oxidation of the cucurbitacin skeleton to produce 11-carbonyl-cucurbitadienol (Zhou et al. 2016). Based on the crystal structure of CYP51 derived from *S. cerevisiae*, Li et al. constructed a homologous model of CYP87D20 with Rosetta and further carried out three rounds of structural and data-driven direct evolutions to modify CYP87D20 (formation of cucurbitadienol-C11 ketone and C20 hydroxyl) into an efficient cucurbitadienol-C11-hydroxylase (Fig. 24).

**Fig. 24:**
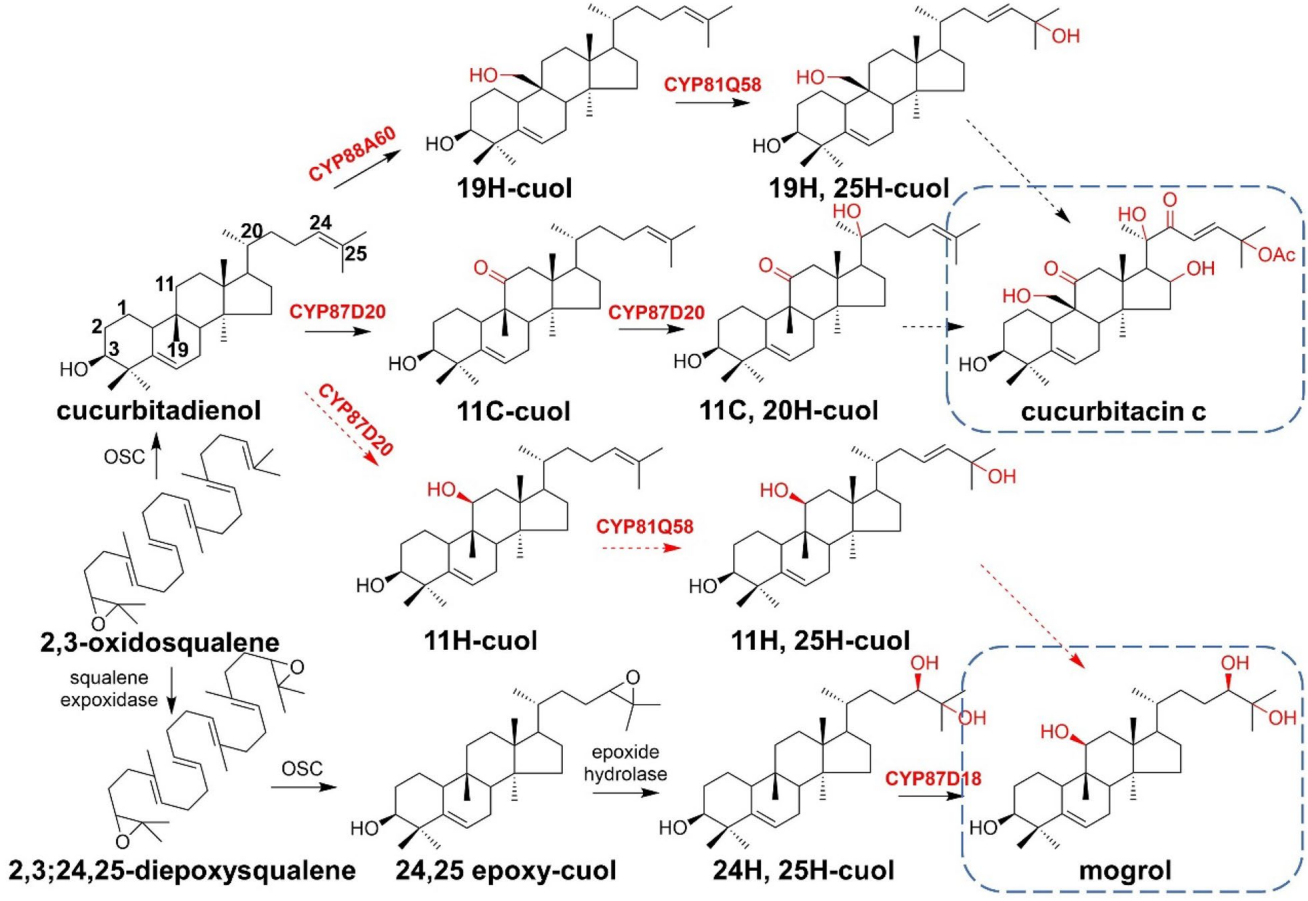
Biosynthesis pathways of CuC and mogrol. Proposed metabolic flux to mogrol is indicated with red arrow. *OSC* oxidosqualene cyclase, *cuol* cucurbitadienol
  In the first round of engineering (R1), amino acids within the range of substrate 6 Å that may affect the specificity of the substrate/product were analyzed. Some mutants produce an increased amount of 11-hydroxyl-cucurbitadienol, as well as a decreased amount of 11-carbonyl-cucurbitadienol or 11-carbonyl-20-hydroxyl-cucurbitadienol. In the second round of engineering (R2), the amino acids around the positive mutants screened by R1 were selected, which further improved the selectivity and activity. In the third round of mutations, 7 mutants from R1 and R2 were selected, and 2 to 3 mutation sites were randomly inserted into these 7 mutants by computer algorithm, and finally more than 27,000 potential mutants were obtained in silico and detected by experiment. After three rounds of modifications and evaluations, Li et al. successfully carries out a single specific hydroxylation at the C-11 position for CYP87D20, thereby increasing the metabolic flux to mogrol (Li et al. 2019).
3. **Manipulate the chemo- and regioselectivity of terpenoid hydroxylases**
  Most plant-derived CYP450s can act on various substrates to generate a spectrum of products. Enzymatic multi-specificity and promiscuity are entirely new propositions in metabolic engineering, and canonical enzyme engineering strategies for modulating the activity of enzymes are largely inapplicable for addressing this phenomenon (Yadav 2014).
  CYP725A4, the first hydroxylase involved in the biosynthetic pathway of taxol, catalyzing taxadiene to form multiple products, including several mono- and di-hydroxylated diterpenoids. Through homology modeling and molecular docking, Edgar et al. selected amino acids within the activation center to construct a mutant library with the aim to improve the yield of di-hydroxylated product. Although most of the mutants did not achieve enhanced selectivity, T380S improved the percentage of 5(12)-oxa-3(11)-cyclo-taxan-10-ol in a variety of products. The presumed production process of this compound is shown in Fig. 25 (Edgar et al. 2017).

**Fig. 25:**
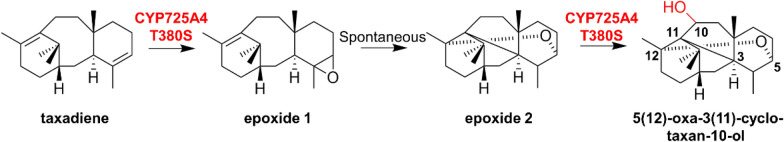
Proposed pathway from taxadiene to 5(12)-oxa-3(11)-cyclo-taxan-10-ol
  CYP72A63 derived from *M. truncatula* has the capability of oxidizing 11-oxo-β-amyrin to produce a range of rare licorice triterpenoids with a low selectivity, including three C-30 oxidized products glycyrrhetol, glycyrrhetaldehyde, and glycyrrhetinic acid, and a C-29 hydroxynated product 29-OH-11-oxo-β-amyrin. Sun et al. identified the key amino acids affecting the specificity of CYP72A63 through homologous modeling and molecular docking. The obtained mutations can specifically convert 11-oxo-β-amyrin to the corresponding hydroxyl, aldehyde, and carboxylic acid, respectively (Fig. 26).

**Fig. 26:**
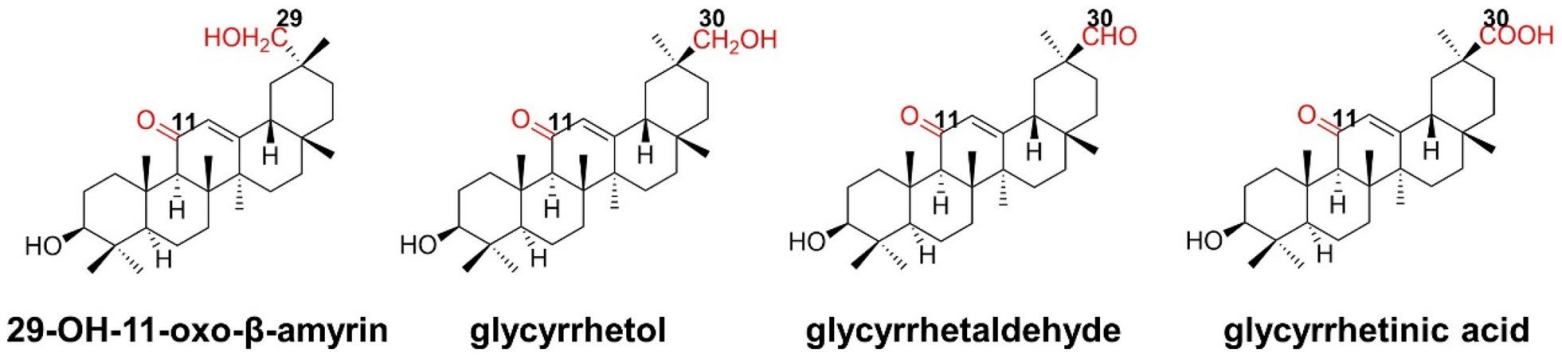
Four kinds of triterpenoids produced by CYP72A63 with 11-oxo-β-amyrin as substrate
  Firstly, substrate 11-oxo-β-amyrin was docked into the active center of CYP72A63. It was found that C-30 and C-29 were all within the catalytic range of heme Fe, while C-30 was closer, which was consistent with the higher oxidation preference at the site of C-30. Secondly, to exploit the consecutive three-step oxidation at C-30, glycyrrhetol was docked with CYP72A63 to reveal a less fitness than 11-oxo-β-amyrin in CYP72A63. Based on the model, it is thus speculated that the hydrophilic hydroxy group at the C-30 position of glycyrrhetol is difficult to enter the active center because of the hydrophobic force of the active site enhanced by the methyl side chain of Thr338.
  After mutating Thr338 to Ser, the side-chain hydroxyl group of Ser points to the active center, increasing the hydrophilicity of the active pocket and enabling glycyrrhetol to be almost completely converted to glycyrrhetinic acid. At the same time, this mutation also makes C-29 farther away from the heme Fe, avoiding the formation of byproduct 29-OH-11-oxo-β-amyrin. Additionally, when Leu509 located in the distal active pocket of CYP72A63 is mutated to Ile, Thr, and Asn, that can significantly hinder the further oxidation of glycyrrhetol, thereby generating glycyrrhetol in high yield (Fig. 27). These mutants constructed based on the homologous models enabled the generation of different licorice-type triterpenoids with high regional and chemical selectivity (Sun et al. 2020).

**Fig. 27:**
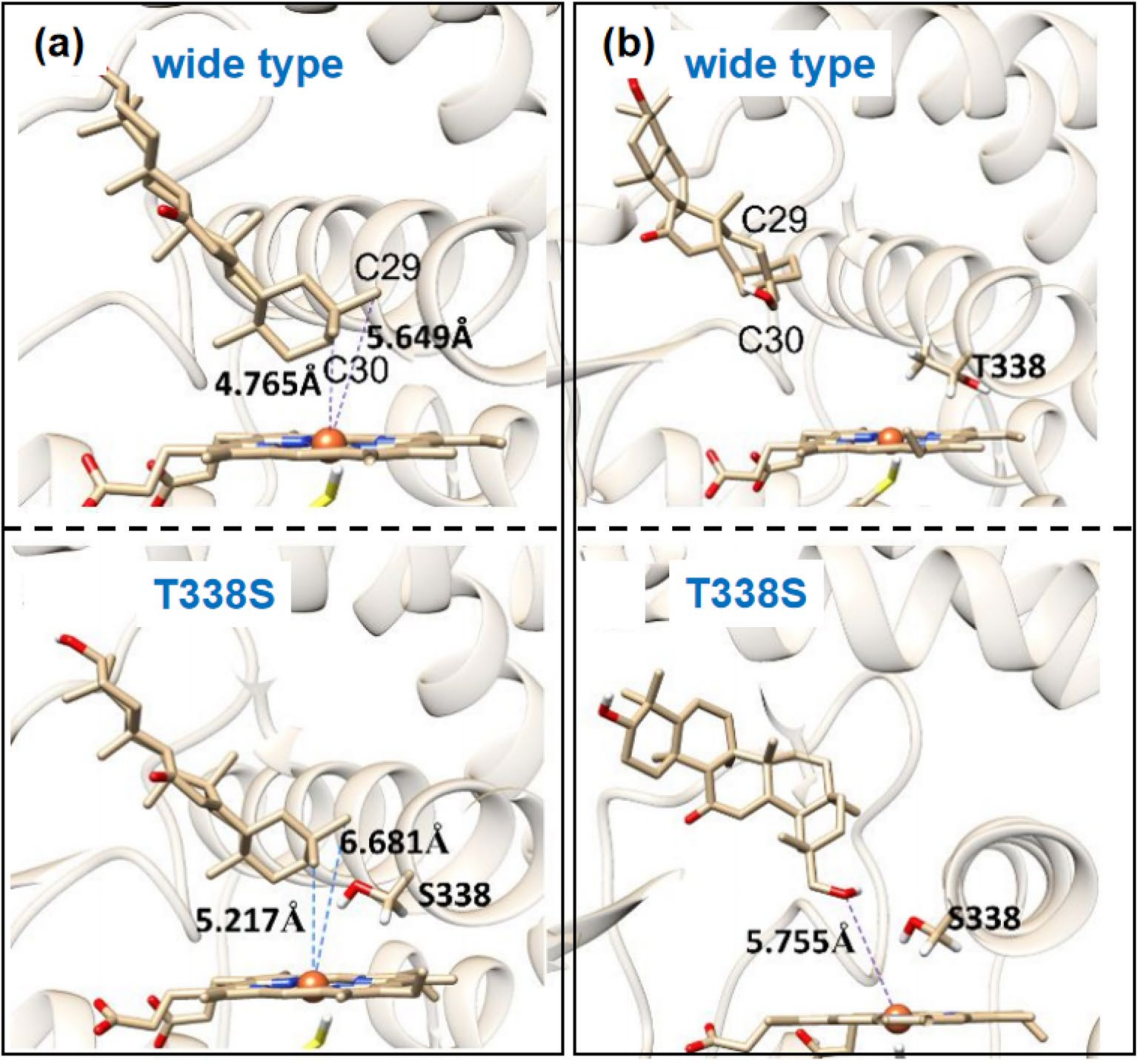
**a** The structure model of CYP72A63 with 11-oxo-β-amyrin; **b** The structure model of CYP72A63 (T338S) with glycyrrhetol

## Summary and outlook

Biocatalytic oxidation equips enormous potential in effectively obtaining bioactive and medicinally relevant terpenoids. Especially, the site-specific and stereo-specific hydroxylation mainly mediated by cytochrome P450 monooxygenases addressed lots of unsolved challenges in total synthesis of natural products by now. A series of monoterpene, sesquiterpene, diterpene, and other terpene hydroxylases have been discovered in plants, animals, and microorganisms through genome mining and cDNA library screening based on homologous sequences, which has made an important contribution to the study of terpenoids biosynthesis.

Additionally, the number of enzymes that have been identified are still relatively limited compared with the natural resources. With advances in sequencing technology, it is anticipated that more new hydroxylases would be discovered and characterized. These hydroxylases usually have low heterologous expression, poor stability, and low catalytic efficiency. Through *N*-terminal modification and redox partner optimization, the expression of P450s in prokaryotic host had been increased apparently. With the development of new technology like cell-free expression system (Walter et al. 2022), it is expected that expression no longer being the bottleneck restricting the further investigation towards terpene hydroxylases.

Finally, most eukaryotic terpene hydroxylase belongs to type II CYP450s, their activity and expression all restricted by the inherent structure and catalytic properties. In view of this, it is also a feasible method to explore and modify other oxygenase superfamilies (Renata 2021; Zwick and Renata 2020), so that they can catalyze corresponding terpene substrates to generate more valuable terpenoids and structural derivatives. Researches on hydroxylases involved in terpenoid biosynthesis is of great significance for promoting the discovery and synthesis of more bioactive medicinal resources.

## Data Availability

Not applicable.
